# HDAC3 maintains oocyte meiosis arrest by repressing amphiregulin expression before the LH surge

**DOI:** 10.1038/s41467-019-13671-8

**Published:** 2019-12-16

**Authors:** Huarong Wang, Han Cai, Xiao Wang, Meiling Zhang, Bingying Liu, Ziqi Chen, Tingting Yang, Junshun Fang, Yanhao Zhang, Wei Liu, Jun Han, Qirui Guo, Hua Zhang, Haibin Wang, Guoliang Xia, Chao Wang

**Affiliations:** 1 State Key Laboratory of Agrobiotechnology, College of Biological Sciences, China Agricultural University, 100193 Beijing, China; 2 Medical College of Xiamen University, 361005 Xiamen, China; 3 Beijing Advanced Innovation Center for Genomics, Biomedical Pioneering Innovation Center, College of Life Sciences, Peking University, 100871 Beijing, China; 4 Shanghai Key Laboratory for Assistant Reproduction and Reproductive Genetics, Center for Reproductive Medicine, Ren Ji Hospital, School of Medicine, Shanghai Jiao Tong University, 200127 Shanghai, China; 5 Reproductive Medical Center, Drum Tower Hospital Affiliated to Nanjing University Medical College, Zhongshan Road 321, 210008 Nanjing, China; 6 Key Laboratory of Ministry of Education for Conservation and Utilization of Special Biological Resources in the Western China, College of Life Science, Ningxia University, 750021 Yinchuan, Ningxia China

**Keywords:** Reproductive disorders, Reproductive disorders

## Abstract

It is known that granulosa cells (GCs) mediate gonadotropin-induced oocyte meiosis resumption by releasing EGF-like factors in mammals, however, the detailed molecular mechanisms remain unclear. Here, we demonstrate that luteinizing hormone (LH) surge-induced histone deacetylase 3 (HDAC3) downregulation in GCs is essential for oocyte maturation. Before the LH surge, HDAC3 is highly expressed in GCs. Transcription factors, such as FOXO1, mediate recruitment of HDAC3 to the amphiregulin (*Areg*) promoter, which suppresses AREG expression. With the LH surge, decreased HDAC3 in GCs enables histone H3K14 acetylation and binding of the SP1 transcription factor to the *Areg* promoter to initiate AREG transcription and oocyte maturation. Conditional knockout of *Hdac3* in granulosa cells in vivo or inhibition of HDAC3 activity in vitro promotes the maturation of oocytes independent of LH. Taking together, HDAC3 in GCs within ovarian follicles acts as a negative regulator of EGF-like growth factor expression before the LH surge.

## Abstract (web summary)

Before ovulation, a surge of luteinizing hormone (LH) triggers the resumption of meiosis in oocytes, which is mediated by EGF-like growth factors. Here, the authors show that HDAC3 inhibits mouse oocyte maturation by negatively regulating the expression of EGF-like factor before the LH surge.

## Introduction

In mammalian follicles, somatic cells and oocytes constitute an intriguing combination that facilitates mutual signaling and chemical communication to orchestrate oocyte meiotic maturation in response to gonadotropin stimulation^1,2^. Fully grown oocytes are generally arrested at prophase of meiosis I by the accumulation of cyclin AMP (cAMP) produced by G-protein-coupled receptor (GPR3/12) signaling to inhibit the activity of maturation-promoting factor^2^. Prior to the luteinizing hormone (LH) surge, follicle-stimulating hormone (FSH) promotes the secretion of natriuretic peptide precursor type C (CNP/NPPC) from mural granulosa cells (mGCs) to maintain oocyte meiosis arrest via the regulation of cyclin GMP (cGMP) produced by cumulus cells^3^. cGMP enters oocytes via gap junctions to evoke meiosis arrest by inhibiting PDE3A activity, which specifically degrades cAMP^4,5^.

When the LH surge occurs, LH decreases NPPC/NPR2 expression levels, thereby blocking cGMP synthesis. Simultaneously, LH stimulates mGCs to secrete epidermal growth factor (EGF)-like growth factors, especially amphiregulin (AREG), epiregulin, and beta-cellulin, which activate EGF receptor (EGFR) signaling in cumulus cells, leading to cGMP degradation in response to PDE5 activation and NPR2 inactivation and resulting in cumulus expansion and oocyte maturation. Finally, LH terminates GC proliferation and induces GC luteinization by activating extracellular signal-regulated kinase 1/2 signaling^6^. The molecular regulatory mechanism of the sex hormone-controlled expression of NPPC and its receptor (NPR2) in response to FSH/LH induction in vivo has been reported^7,8^. However, the signaling pathway downstream of LH receptor involved in regulating EGF-like growth factor expression and release has not yet been characterized.

Although epigenetic modifications are important for germ cell development^1,9–13^, few reports have assessed the effects of epigenetic modifications in somatic follicular cells on gonadotropin-induced oocyte maturation. One exception is a study showing that the level of histone deacetylase 3 (HDAC3) was increased in GCs retrieved from polycystic ovary syndrome patients who experienced in vitro fertilization (IVF) failure compared with those from patients who experienced successful IVF^14^. HDAC3 belongs to the class I family of HDACs that regulates gene expression by acetylating lysine residues in histone tails^15,16^. Therefore, we hypothesize that HDAC3 levels in GCs may be associated with oocyte maturation quality. In addition, it remains unknown whether this association is related to gonadotropin induction.

Here we demonstrate that HDAC3 inhibits AREG expression through H3K14 deacetylation prior to the LH surge. Conditional knockout and inhibition of HDAC3 in GCs increase AREG expression and result in LH-independent oocyte maturation in mice.

## Results

### HDAC3 negatively regulated oocyte maturation

**Fig. 1:**
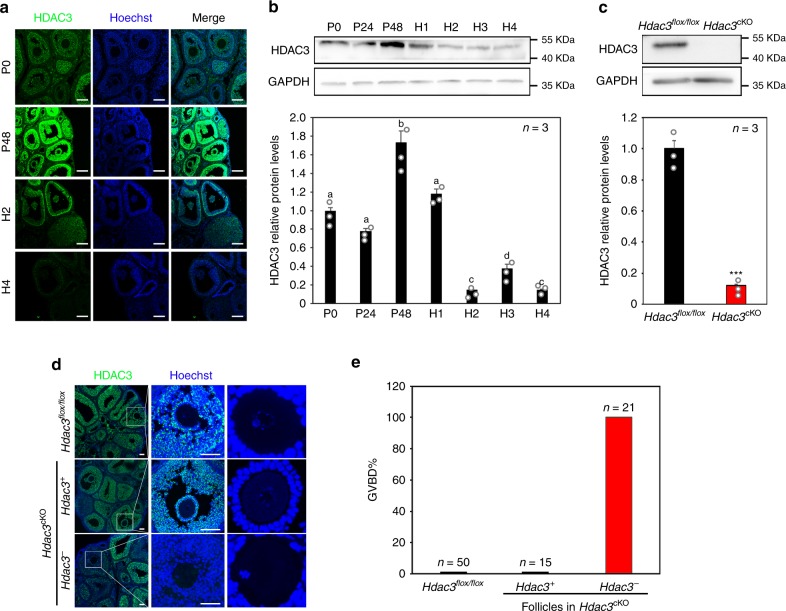
HDAC3 negatively regulated oocyte maturation. **a** HDAC3 (green) levels in GCs decreased dramatically in response to hCG stimulation. Nuclei were dyed with Hoechst (blue). Scale bar = 100 µm. *n* = 3 biologically independent experiments. **b** HDAC3 protein levels were upregulated by PMSG and downregulated by hCG in a time-dependent manner. P48: 48 h after PMSG treatment, equivalent to 48 h after the FSH surge. H1–H4: 1–4 h after hCG treatment, equivalent to 1–4 h after the LH surge. *n* = 3 biologically independent experiments. Data are presented as mean ± SEM, and different letters (a–d) indicate significant differences among groups (two-sided ANOVA test), *P* (a, b) < 0.05, *P* (a, c) < 0.01, *P* (a, d) < 0.01, *P* (b, c) < 0.001, *P* (b, d) < 0.01, *P* (c, d) < 0.05. **c** *Hdac3* knockout efficiency was examined and quantified by western blotting analysis. *n* = 3 biologically independent experiments. Data are presented as mean ± SEM. Asterisks (*) indicate significant differences at ***P = 0.00007 determined by the two-sided *t* test. **d** HDAC3 (green) immunostaining in the different mouse models revealed oocyte maturation independent of LH in follicles lacking *Hdac3* expression (*Hdac3*^-^) in GCs, whereas oocytes in *Hdac3*-positive (*Hdac3*^+^) follicles remained in the GV stage, similar to the situation in *Hdac3*^*flox/flox*^ mice. Scale bar = 100 µm. Nuclear DNA in indicated in blue (Hoechst). **e** Oocyte maturation (GVBD) rates in follicles with or without HDAC3 expression in the different mouse genotypes. Source data are provided as a Source Data file.

To investigate the physiological role of HDACs during oocyte maturation in mice, the expression patterns of all of the class I HDACs in growing follicles induced by gonadotropins were studied. HDAC3 was the only HDAC in GCs that was upregulated by FSH but downregulated by LH in vivo and in vitro (Fig. 1a, b; Supplementary Fig. 1a–c), demonstrating its pivotal role in response to gonadotropin induction during oocyte maturation.

Then, to investigate the precise ovarian function of HDAC3, a mouse model with GC-specific deletion of *Hdac3* was produced by crossing *Hdac3*^*flox/flox*^ female mice with *Foxl2-Cre*ER^T2^ transgenic mice plus *Hdac3*^*flox/flox*^ male mice, yielding *Hdac3*^*flox/flox*^; *Foxl2-Cre*ER^T2^ (*Hdac3*^cKO^) female mice^17^. Eight days after the respective intraperitoneal (i.p.) injection of tamoxifen in 18-day-old *Hdac3*^cKO^ female mice and *Hdac3*^*flox/flox*^ female mice (as a negative control), the western blotting results showed that HDAC3 protein levels in GCs were markedly decreased in *Hdac3*^cKO^ mice compared with the control (Fig. 1c).

To clarify whether the deletion of HDAC3 in GCs correlates with oocyte maturation, pregnant mare serum gonadotropin (PMSG) was respectively injected into *Hdac3*^cKO^ and *Hdac3*^*flox/flox*^ mice to promote follicle development 6 days after tamoxifen administration. Although *Hdac3* depletion was incomplete due to limitations of the mouse model, we found that the oocytes in *Hdac*3-deleted follicles were premature 44 h after PMSG injection, while the oocytes in ovarian follicles of *Hdac3*^*flox/flox*^ mice and in HDAC3-positive follicles of *Hdac3*^cKO^ mice, similar to the oocytes in wild-type (WT) mice and the *Foxl2-Cre*ER^T2^ transgene but not carrying the *floxed* allele mice, were still at the germinal vesicle (GV) stage (Fig. 1d, e; Supplementary Fig. 2a–c). These results indicated that *Hdac3* deletion results in premature oocyte maturation before the LH surge begins.

To examine whether *Hdac3* depletion affects ovarian follicle survival and follicular atresia, which probably result in oocyte GV breakdown (GVBD), we performed quantitative polymerase chain reaction (qPCR) to determine the *Bcl2/Bax* ratio and terminal deoxynucleotidyl transferase-mediated dUTP-fluorescein nick end labeling (TUNEL) assays to evaluate GC survival. The results showed that the *Bcl2*/*Bax* ratio was normal in *Hdac3*^cKO^ mouse ovarian GCs (Supplementary Fig. 2d). Similarly, TUNEL assays showed that oocyte GVBD in *Hdac3* knockout ovarian follicles was not caused by GC apoptosis or follicular atresia (Supplementary Fig. 2e). Therefore, the GC-specific knockout of *Hdac3* did not affect the development or survival of GCs and follicles. Furthermore, LH can terminate ovarian GC proliferation, and we found that *Hdac3* depletion also prohibited GC proliferation, as indicated by proliferating cell nuclear antigen staining (Supplementary Fig. 2f). These results indicated that *Hdac3* depletion in ovarian GCs probably mimics the effects of LH on oocyte maturation and GC proliferation.

### HDAC3 repressed AREG expression in GCs before the LH surge

**Fig. 2:**
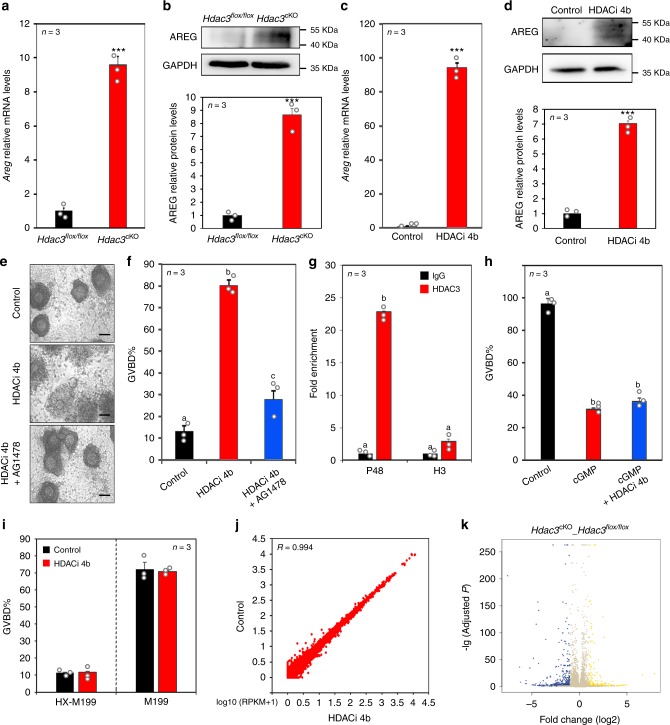
HDAC3 repressed AREG expression in GCs before the LH surge. **a** Quantitative real-time PCR revealed *Areg* expression in *Hdac3*^*flox/flox*^ and *Hdac3*^cKO^ GCs after PMSG treatment. Data are presented as mean ± SEM. Asterisks (*) indicate significant differences at ***P = 0.000003 determined by the two-sided *t* test. **b** AREG levels in *Hdac3*^*flox/flox*^ and *Hdac3*^cKO^ GCs were quantified by western blotting analysis. *n* = 3 biologically independent experiments. Data are presented as mean ± SEM. Asterisks (*) indicate significant differences at ***P = 0.0009 determined by the two-sided *t* test. **c** Quantitative real-time PCR showed that inhibiting HDAC3 increased *Areg* expression in GCs in vitro. HDACi 4b, an HDAC3 inhibitor. Data are presented as mean ± SEM. Asterisks (*) indicate significant differences at ***P = 5.15E−5 determined by the two-sided *t* test. **d** AREG levels in control and HDACi 4b-treated GCs were quantified by western blotting analysis. *n* = 3 biologically independent experiments. Data are presented as mean ± SEM. Asterisks (*) indicate significant differences at ***P = 3.27E−5 determined by the two-sided *t* test. **e** Representative images demonstrate the effect of GC-derived HDAC3 on cumulus cell expansion within in vitro cultured COCs. Scale bar = 100 µm. *n* = 3 biologically independent experiments. **f** Oocyte maturation (GVBD) rates were significantly improved by HDACi 4b, and this effect was reversed by AG1478. AG1478, EGFR inhibitor. *n* = 3 biologically independent experiments. Data are presented as mean ± SEM, and different letters (a–c) indicate significant differences among groups (two-sided ANOVA and Holm–Šidák test), *P* (a, b) < 0.001, *P* (a, c) < 0.05, *P* (b, c) < 0.001. **g** ChIP-qPCR analysis of HDAC3 at the *Areg* promoter (−343 to −150) before and after the LH surge. *n* = 3 biologically independent experiments. Data are presented as mean ± SEM, and different letters (a–b) indicate significant differences among groups (two-sided ANOVA and Holm–Šidák test), *P* (a, b) < 0.001. **h** HDACi 4b was unable to rescue 8-Br-cGMP-mediated inhibition of oocyte maturation (GVBD). Data are presented as mean ± SEM, and different letters (a–b) indicate significant differences among groups (two-sided ANOVA and Holm–Šidák test), *P* (a, b) < 0.001. **i** HDACi 4b had no direct effect on denuded oocyte meiotic maturation. **j** Scatter plot comparison of oocyte transcripts between the control and HDAC3 inhibitor groups revealed that HDACi 4b had no effect on oocyte developmental competence. Pearson statistical test used for statistical analysis*.* **k** Volcano plot of transcriptomic analysis of GCs isolated from *Hdac3*^*flox/flox*^ and *Hdac3*^cKO^ ovaries. Source data are provided as a Source Data file.

Given that NPPC and EGF-like growth factors are the two types of molecules secreted by GCs that are critical in the regulation of oocyte meiosis progression in response to gonadotropin induction^3,6^, two possible pathways may be responsible for the effects of *Hdac3* deletion on oocyte maturation. Specifically, *Hdac3* deletion in GCs may either downregulate NPPC expression to relieve oocytes from the inhibitory environment or upregulate EGF-like growth factors to re-initiate oocyte meiosis. Interestingly, the mRNA and protein levels of only AREG, one of the most important EGF-like growth factors that responds to LH induction, were significantly increased in the ovaries of *Hdac3*^cKO^ mice 40 h after PMSG induction (Fig. 2a, b; Supplementary Fig. 3a). These findings indicate that AREG likely mediates HDAC3 action in oocyte maturation.

To confirm the HDAC3-mediated regulation of AREG expression, follicle cultures in vitro were treated with HDACi 4b, a novel, synthetic, specific inhibitor of HDAC1/3^18^. Consistent with the effects of *Hdac3* knockout in vivo, AREG protein and mRNA levels were significantly elevated by HDACi 4b (Fig. 2c, d). Moreover, cumulus expansion and oocyte meiosis resumption were induced by HDACi 4b, while these effects were blocked by the EGFR inhibitor AG1478, confirming the role of AREG in the effects of HDAC3 on oocyte maturation (Fig. 2e, f). Furthermore, chromatin immunoprecipitation (ChIP) analysis showed that, before the LH surge, more HDAC3 protein was recruited to the *Areg* promoter, while 3 h after human chorionic gonadotropin (hCG) treatment (equivalent to 3 h after the LH surge), HDAC3 recruitment to the *Areg* promoter was significantly decreased (Fig. 2g). HDAC1 was unable to bind the *Areg* promoter (Supplementary Fig. 3b). Therefore, we concluded that HDAC3 can specifically bind to the *Areg* promoter region (−343, −150) before the LH surge.

Furthermore, cumulus cells in cumulus oocyte complexes (COCs) were reported to express AREG upon indirect induction by the LH surge in vivo and upon direct induction by FSH in vitro during oocyte maturation^19,20^, suggesting that AREG expression in cumulus cells is also essential for oocyte maturation. To examine whether HDAC3 regulates AREG expression in cumulus cells, we examined *Areg* expression levels after mouse COCs were cultured with or without HDACi 4b in vitro in NPPC-M199 medium. The results showed that HDACi 4b was able to promote oocyte maturation in COCs by directly inducing AREG expression in cumulus cells (Supplementary Fig. 4). Collectively, the data indicated that HDAC3 is a negative regulator of AREG expression that is essential for gonadotropin-induced oocyte maturation.

It is well known that cGMP produced by NPR2 is essential for oocyte meiosis arrest, which affects upon diffusion via gap junctions^2^. AREG activates EGFR signaling in cumulus cells, which suppresses the guanylyl cyclase activity of NPR2 and activates PDE5 activity to decrease cGMP levels^2,21^. To examine whether the HDAC3 inhibitor induces oocyte maturation by changing cGMP levels in cumulus cells, we cultured COCs with 8-Br-cGMP alone or in combination with HDACi 4b in M199 medium. The results showed that, while most of the oocytes in the control group resumed meiosis, the oocytes in the group treated with 8-Br-cGMP alone arrested at the GV stage; however, the oocytes in the group treated with HDACi 4b and 8-Br-cGMP were unable to resume meiosis, with most remaining at the GV stage (Fig. 2h). Therefore, we concluded that HDAC3 deficiency induced oocyte maturation by regulating the levels of cGMP, which is well known to diffuse from cumulus cells to oocytes through gap junctions^2^.

An important question is whether HDACi 4b directly affects denuded oocyte maturation and developmental competence. Therefore, the effects of HDACi 4b on denuded oocytes were examined in HX-M199 and M199 media. In HX-M199 medium, most of the oocytes in the control and HDACi 4b-treated groups were arrested in the GV stage (Fig. 2i; Supplementary Fig. 5). In M199 medium, most of the oocytes in the control and HDACi 4b-treated groups resumed meiosis (Fig. 2i; Supplementary Fig. 5). These results demonstrated that HDACi 4b has no direct effect on oocyte meiotic maturation.

Furthermore, to detect whether HDACi 4b affects developmental competence, we performed transcriptome analysis of all the mature oocytes in the control and HDACi 4b-treated groups. The correlation coefficient between global transcripts in the control and HDACi 4b-treated groups was >0.99, and no differentially expressed genes were found (Fig. 2j and Supplementary Data 1), indicating that HDACi 4b has no effect on oocyte developmental competence.

Although HDAC3 and HDACi 4b controlled oocyte maturation by regulating AREG expression was proved in this study, other oocyte maturation-related factors regulated by HDAC3 remained unidentified. Therefore, we performed RNA-seq analysis of ovarian GCs from *Hdac3*^cKO^ and *Hdac3*^*flox/flox*^ mice and of GCs treated with HDACi 4b or control. Compared with ovarian GCs from *Hdac3*^*flox/flox*^ mice, those from *Hdac3*^cKO^ mice showed the differential expression of as many as 233 transcripts, with 78 upregulated transcripts, including *Areg*, and 155 downregulated transcripts (Fig. 2k and Supplementary Data 2). The genes with the most pronounced changes in expression are shown in Supplementary Fig. 6a. Impressively, more general transcriptome changes were detected in GCs treated with HDACi 4b in vitro than in those treated with the control: 2105 transcripts were upregulated and 1301 transcripts were downregulated (Supplementary Fig. 6c and Supplementary Data 3). The Gene Ontology (GO) terms associated with the altered transcripts were also analyzed (Supplementary Fig. 6b, d).

### H3K14ac mediated HDAC3 and LH action regarding *Areg* expression

**Fig. 3:**
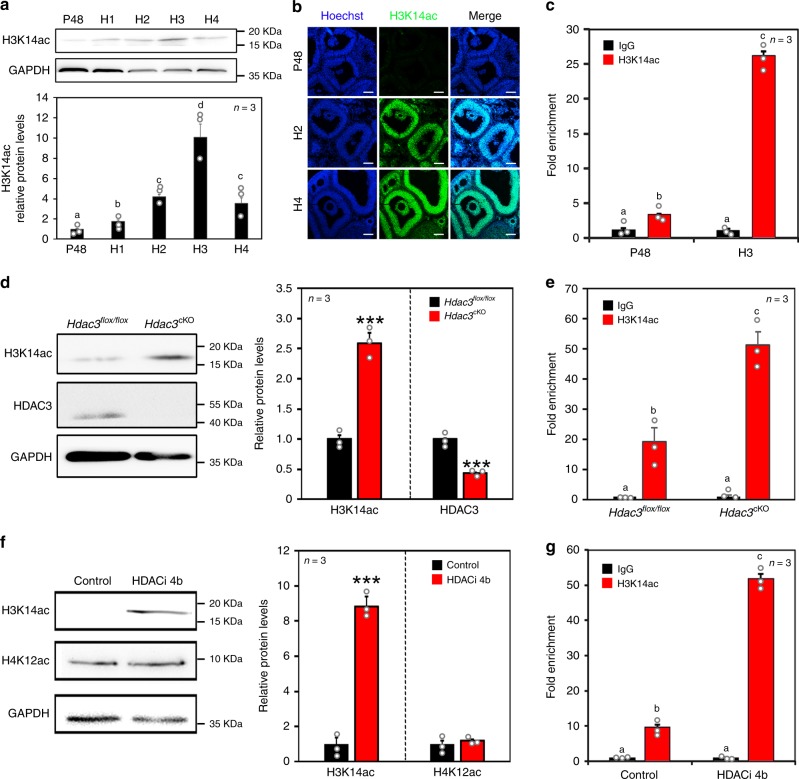
H3K14ac mediated the effects of HDAC3 and LH on *Areg* expression. **a** H3K14ac levels were increased by hCG in a time-dependent manner. *n* = 3 biologically independent experiments. Data are presented as mean ± SEM, and different letters (a–d) indicate significant differences among groups (two-sided ANOVA and Holm–Šidák test), *P* (a, b) < 0.01, *P* (a, c) < 0.01, *P* (a, d) < 0.001, *P* (b, c) < 0.05, *P* (b, d) < 0.01, *P* (c, d) < 0.05. **b** Immunostaining revealed that LH increased H3K14ac (green) levels in GCs in vivo. Nuclei were dyed with Hoechst (blue). Scale bar = 100 µm. *n* = 3 biologically independent experiments. **c** ChIP-qPCR analysis of H3K14ac at the *Areg* promoter (−343 to −150) before and after the LH surge. P48: 48 h after PMSG treatment. H1–H4: 1–4 h after hCG treatment. Data are presented as mean ± SEM, and different letters (a–c) indicate significant differences among groups (two-sided ANOVA and Holm–Šidák test), *P* (a, b) < 0.01, *P* (a, c) < 0.001, *P* (b, c) < 0.001. **d** H3K14ac levels in *Hdac3*^*flox/flox*^ and *Hdac3*^cKO^ GCs were quantified by western blotting analysis. *n* = 3 biologically independent experiments. Data are presented as mean ± SEM. Asterisks (*) indicate significant differences at ***P < 0.001 determined by the two-sided *t* test. **e** ChIP-qPCR analysis of H3K14ac at the *Areg* promoter in *Hdac3*^*flox/flox*^ and *Hdac3*^cKO^ GCs. Data are presented as mean ± SEM, and different letters (a–c) indicate significant differences among groups (two-sided ANOVA and Holm–Šidák test), *P* (a, b) < 0.001, *P* (a, c) < 0.001, *P* (b, c) < 0.05. **f** Western blotting analysis showed that HDACi 4b (HDAC3 inhibitor) substantially increased H3K14ac levels. *n* = 3 biologically independent experiments. Data are presented as mean ± SEM. Asterisks (*) indicate significant differences at ***P < 0.001 determined by the two-sided *t* test. **g** ChIP-qPCR analysis of H3K14ac at the *Areg* promoter in control and HDACi 4b-treated GCs. Data are presented as mean ± SEM, and different letters (a–c) indicate significant differences among groups (two-sided ANOVA and Holm–Šidák test), *P* (a, b) < 0.001, *P* (a, c) < 0.001, *P* (b, c) < 0.001. Source data are provided as a Source Data file.

It is well known that HDAC3 packages chromatin into repressed domains via its HDAC activity^22,23^. To identify which histone lysine residues are regulated by HDAC3 in GCs, most of the histone acetylation modifications in GCs during LH/hCG induction were assessed (Supplementary Fig. 7a, b). The results showed that histone 3 lysine 14 acetylation (H3K14ac) was markedly increased by LH/hCG induction in vivo and in vitro (Fig. 3a, b; Supplementary Fig. 7c, d). ChIP analysis demonstrated that H3K14ac levels at the *Areg* promoter were substantially increased in GCs induced by LH/hCG (Fig. 3c), suggesting that H3K14ac probably mediates the actions of LH/hCG on target gene expression.

Furthermore, to elucidate whether HDAC3 inhibits AREG expression by repressing H3K14ac, GCs from HDAC3 knockout mouse ovaries or HDACi 4b-treated follicles were examined. H3K14ac levels were significantly increased in ovarian GCs from *Hdac3*^cKO^ mice compared with *Hdac3*^*flox/flox*^ mice (Fig. 3d). Furthermore, ChIP analysis revealed that H3K14ac levels at the *Areg* promoter were obviously increased in GCs from *Hdac3*^cKO^ mouse ovaries compared with *Hdac3*^*flox/flox*^ mouse ovaries (Fig. 3e). Correspondingly, H3K14ac levels were much higher in HDACi 4b-treated GCs than in control GCs (Fig. 3f). ChIP analysis showed that HDACi 4b increased H3K14ac levels at the *Areg* promoter in GCs in vitro (Fig. 3g). Therefore, H3K14ac is key for the regulation of AREG expression by both LH and HDAC3. The results unanimously confirmed our hypothesis that HDAC3 decreases H3K14ac levels in vivo and in vitro before LH induction.

### FOXO1 potentially recruited HDAC3 to the *Areg* promoter

**Fig. 4:**
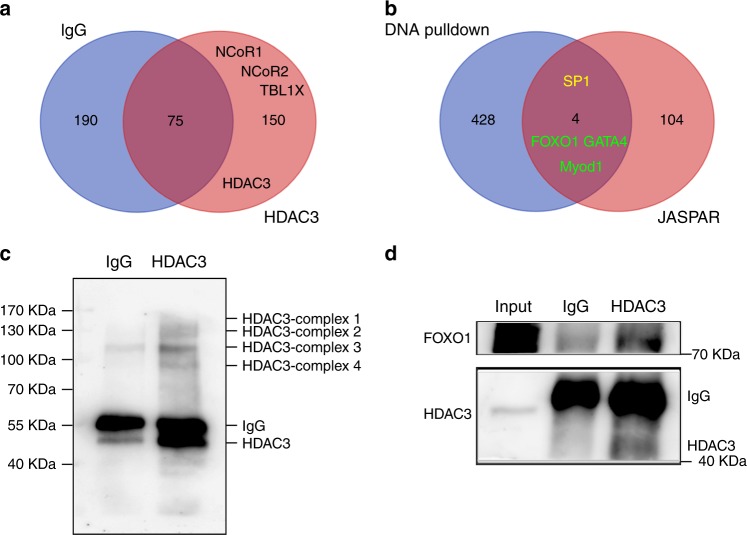
FOXO1 potentially recruited HDAC3 to the *Areg* promoter. **a** Venn diagram of HDAC3 partners between the HDAC3-IP group and the negative IgG group revealed that HDAC3 could interact with the core members of the NCoR complex. **b** Venn diagram of potential transcription factors that bind the *Areg* promoter between the DNA pulldown data and JASPAR data. Among these transcription factors, FOXO1, GATA4, and Myod1 (green), but not SP1 (yellow), were identified as HDAC3 interactors. **c** Cross-linking Co-IP coupled with western blotting analysis of HDAC3 showed the captured HDAC3 complexes at the chromatin. *n* = 3 biologically independent experiments. **d** Reverse cross-linking Co-IP coupled with western blotting analysis of HDAC3 demonstrated the interaction between HDAC3 and FOXO1. *n* = 3 biologically independent experiments. Source data are provided as a Source Data file.

Among the class I HDAC family, HDAC3 is the only member that functions through interacting with NCoR1/2 and TBL1X/TBL1XR, forming the core of the NCoR transcriptional repressor complex through its deacetylase activation domain^24^. In line with this finding, we performed coimmunoprecipitation (Co-IP) of HDAC3 coupled with mass spectrometry (MS) to identify factors that bind HDAC3 to repress *Areg* expression. The core members of the NCoR complex, NCoR1, NCoR2, and TBL1X, were all identified in the HDAC3 antibody group compared with the immunoglobulin G (IgG) group (Fig. 4a; Supplementary Data 4 and 5).

Another important unknown is the transcription factor that recruits the HDAC3-NCoR complex to the *Areg* promoter. We performed DNA pulldown coupled with MS analysis and searched for predicted transcription factor-binding sites using the JASPAR database. By comparing the proteins captured by *Areg* promoter DNA with the transcription factor-binding data predicted by JASPAR, four candidate transcription factors, namely, SP1, FOXO1, GATA4, and Myod1, were identified (Fig. 4b; Supplementary Data 6 and 7). However, none of these transcription factors were identified by HDAC3 Co-IP (Fig. 4a; Supplementary Data 4 and 5), indicating that they potentially interact with the HDAC3-NCoR complex rather than with HDAC3 directly. Therefore, we performed cross-linking Co-IP followed by MS to examine all of the proteins directly or indirectly interacting with HDAC3 on chromatin^25^. Western blotting analysis of cross-linking Co-IP against HDAC3 showed that numerous HDAC3 complexes were successfully captured in the HDAC3 IP group compared to the negative IgG group (Fig. 4c). As expected, MS data analysis identified many transcription factors among the HDAC3 interactors on chromatin, including the enrichment of NCoR complex components (Supplementary Data 8). However, only FOXO1, GATA4, and Myod1, but not SP1, were found among the HDAC3 interactors, indicating that these three transcription factors potentially interact with the HDAC3 complex at the *Areg* promoter (Fig. 4b; Supplementary data 8). Furthermore, the interaction between FOXO1 and HDAC3 was confirmed by reverse cross-linking Co-IP coupled with western blotting (Fig. 4d). Therefore, we concluded that one or more of the FOXO1, GATA4, and Myod1 transcription factors are potentially recruited by HDAC3 to the *Areg* promoter before the LH surge.

### HDAC3 and LH affected *Areg* expression by regulating SP1 recruitment to the *Areg* promoter

**Fig. 5:**
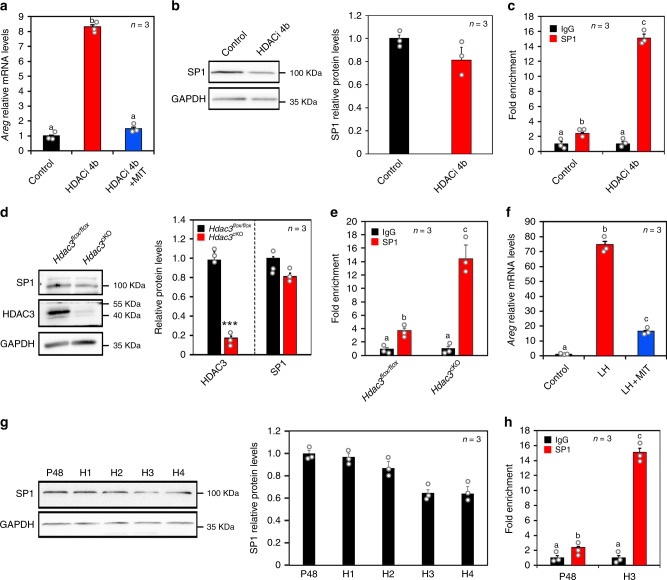
HDAC3 and LH affected *Areg* expression by regulating SP1 recruitment to the *Areg* promoter. **a** Inhibition of SP1 reversed the HDACi 4b-mediated induction of *Areg* expression. MIT, SP1 inhibitor. Data are presented as mean ± SEM, and different letters (a–b) indicate significant differences among groups (two-sided ANOVA and Holm–Šidák test), *P* (a, b) < 0.001. **b** Western blotting analysis revealed that inhibition of HDAC3 did not affect SP1 expression. HDACi 4b, HDAC3 inhibitor. *n* = 3 biologically independent experiments. Data are presented as mean ± SEM. **c** ChIP-qPCR analysis of SP1 at the *Areg* promoter (−343 to −150) in control and HDACi 4b-treated GCs. Data are presented as mean ± SEM, and different letters (a–c) indicate significant differences among groups (two-sided ANOVA and Holm–Šidák test), *P* (a, b) < 0.05, *P* (a, c) < 0.001, *P* (b, c) < 0.001. **d** SP1 levels in *Hdac3*^*flox/flox*^ and *Hdac3*^cKO^ GCs were quantified by western blotting analysis. *n* = 3 biologically independent experiments. Data are presented as mean ± SEM. Asterisks (*) indicate significant differences at ***P < 0.001 determined by the two-sided *t* test. **e** ChIP-qPCR analysis of SP1 at the *Areg* promoter in *Hdac3*^*flox/flox*^ and *Hdac3*^cKO^ GCs. Data are presented as mean ± SEM, and different letters (a–c) indicate significant differences among groups (two-sided ANOVA and Holm–Šidák test), *P* (a, b) < 0.05, *P* (a, c) < 0.001, *P* (b, c) < 0.001. **f** Inhibition of SP1 reversed the LH-mediated induction of *Areg* expression. Data are presented as mean ± SEM, and different letters (a–c) indicate significant differences among groups (two-sided ANOVA and Holm–Šidák test), *P* (a, b) < 0.001, *P* (a, c) < 0.001, *P* (b, c) < 0.001. **g** Western blotting analysis revealed that LH did not affect the SP1 expression in vivo. *n* = 3 biologically independent experiments. Data are presented as mean ± SEM. **h** ChIP-qPCR analysis of SP1 at the *Areg* promoter before and after the LH surge. P48: 48 h after PMSG treatment. H1–H4: 1–4 h after hCG treatment. Data are presented as mean ± SEM, and different letters (a–c) indicate significant differences among groups (two-sided ANOVA and Holm–Šidák test), *P* (a, b) < 0.05, *P* (a, c) < 0.001, *P* (b, c) < 0.001. Source data are provided as a Source Data file.

Histone acetylation induces gene expression by causing chromatin unfolding and increasing transcription factor access^15^; thus we aimed to identify the transcription factors that bind to the *Areg* promoter and initiate transcription upon removal of HDAC3. We found that AREG expression induced by HDAC3 inhibition was reversed by mithramycin A (MIT), a specific inhibitor of the transcription factor SP1 (Fig. 5a), suggesting that SP1 is involved in *Areg* transcription after HDAC3 downregulation. Although HDACi 4b and *Hdac3* knockout had no effect on SP1 expression in vitro (Fig. 5b) or in vivo (Fig. 5d), respectively, ChIP analysis revealed increased SP1 protein recruitment to the HDAC3-binding site on the *Areg* promoter in HDACi 4b-treated GCs compared with control cells (Fig. 5c). Correspondingly, *Hdac3* deletion in ovarian GCs significantly increased SP1 occupancy at the *Areg* promoter (Fig. 5e). Collectively, the loss of HDAC3 and the acetylation of H3K14 promoted SP1 binding to the *Areg* promoter.

To investigate the role of SP1 under more physiological conditions, we examined the effect of SP1 on LH-induced AREG expression. MIT notably reversed the high AREG levels induced by LH (Fig. 5f), suggesting that SP1 is involved in *Areg* transcription induced by LH. Consistently, SP1 protein levels in GCs were unaffected by LH during oocyte maturation (Fig. 5g), whereas ChIP analysis revealed that LH strongly promoted SP1 recruitment to the HDAC3-binding site on the *Areg* promoter (Fig. 5h). Taken together, these data indicated that LH induces H3K14ac and increases SP1 access to the *Areg* promoter.

### The HDAC3 inhibitor improved mouse oocyte maturation and developmental competence

**Fig. 6:**
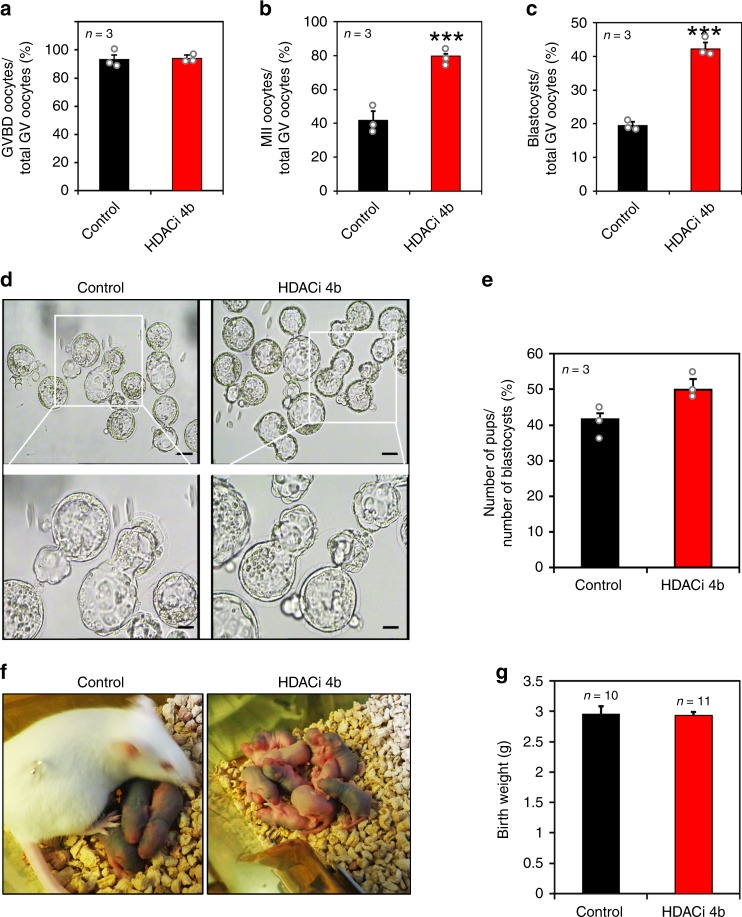
The HDAC3 inhibitor improved mouse oocyte maturation and developmental competence. **a** The GVBD ratios were similar in the control and HDACi 4b groups. HDACi 4b, HDAC3 inhibitor. **b** More MII-stage oocytes were present in the HDACi 4b group than in the control group. Data are presented as mean ± SEM. Asterisks (*) indicate significant differences at ***P = 0.0002 determined by the two-sided *t* test. **c** The blastocyst rate of all GV stage oocytes was considerably increased in the HDACi 4b group compared with the control group. Data are presented as mean ± SEM. Asterisks (*) indicate significant differences at ***P = 0.0006 determined by the two-sided *t* test. **d** Representative images show blastocysts obtained from the control and HDACi 4b groups. Scale bars = 100 µm and 40 µm (enlarged). *n* = 3 biologically independent experiments. **e**, **f** Blastocysts from the control and HDACi 4b groups were transferred into surrogate mice. **e** Birth rates of the control and HDACi 4b groups. **f** Representative images depicting the pups born in the control and HDACi 4b groups. **g** The birth weights of the pups in the control and HDACi 4b groups were similar. The data are presented as the mean ± SEM. Source data are provided as a Source Data file.

In light of our findings demonstrating that the inhibition of GC-derived HDAC3 induces oocyte maturation independent of LH action, we assessed whether HDAC3 is a potential target to improve the developmental capacity of oocytes undergoing in vitro maturation (IVM) independent of LH. Briefly, HDACi 4b was applied to cultured mouse COCs with mGC feeders, which is known as the HDACi 4b-GC feeder system. Although the oocyte GVBD rate of COCs was similar between the control and HDACi 4b groups (93% vs. 94%, respectively; *P* > 0.05) (Fig. 6a), the ratio of metaphase II (MII) oocytes was 1.9-fold higher in the HDACi 4b group than in the control group (85% vs. 45%, *P* < 0.01) (Fig. 6b), demonstrating that our system promoted the GV to MII transition in oocytes.

Then oocyte developmental capacity was further evaluated. In brief, all the MII-stage oocytes obtained from the IVM system were used for IVF. After 4 days of in vitro culture (IVC) of fertilized eggs, up to 2.2-fold more blastocysts were obtained from the HDACi 4b group than from the control group (Fig. 6c, d). The embryo transfer assay demonstrated that the blastocysts in the HDACi 4b group could undergo full pregnancy and even produce slightly more pups, although the difference in pup number was not statistically significant (Fig. 6e, f). Interestingly, the body weights of the pups in the control and HDACi 4b groups at 3 days after birth were similar (Fig. 6g). Collectively, by targeting HDAC3, our IVM system promotes oocyte nuclear and cytoplasmic development competence independent of LH.

## Discussion

Physiologically, the final stage of oocyte maturation requires close cooperation between oocytes and GCs within the follicles^25^. Consistent with this finding, LH response failure leads to failed oocyte maturation and ovulation, and this response is related to abnormal gene expression in GCs^1,9,26–29^. It is therefore reasonable to hypothesize that an essential inhibitory mechanism in GCs may exist that restrains gene expression important for resuming oocyte maturation before the LH induction. The inability to repress the expression of these genes leads to the gonadotropin-independent resumption of meiosis, which interrupts the synchrony between oocyte maturation and ovulation and compromises female fertility^3^. Surprisingly, this study revealed an epigenetic modification mechanism in GCs that histone acetylation by HDAC3 is essential for gonadotropin-induced oocyte maturation (Supplementary Fig. 8).

EGF-like growth factors mediate the effect of LH on oocyte maturation^30^. Among these factors, AREG is the most abundant and important protein^26^. The *Areg* knockout mouse has severely impaired fertility^31^, and AREG levels in follicular fluid are positively correlated with the number of available embryos in humans^32^. However, given the significant differences in bioactivity between endogenous and recombinant AREG, the concentration of recombinant AREG necessary to promote cumulus expansion and oocyte maturation in vitro is approximately 20-fold higher than that detected in follicular fluid^26^. Therefore, employing GC-secreted AREG to improve oocyte maturation in vitro may be a more efficient and physiological strategy. Here AREG expression in both GCs and cumulus cells is essential for oocyte maturation under physiological conditions^19^. HDAC3 regulates AREG expression in both GCs and cumulus cells (Fig. 2a–d; Supplementary Fig. 4a, b). Combined with the result that the LH surge decreased HDAC3 levels in GCs and cumulus cells (Fig. 1a, b; Supplementary Fig. 1b, c), LH decreases HDAC3 protein levels in mGCs directly and in cumulus cells indirectly, resulting in increased AREG expression levels in both mGCs and cumulus cells and oocyte maturation. However, AREG may not be the sole target of HDACi 4b; supplementing the IVM medium with AG1478, a specific inhibitor of the AREG receptor (EGFR), did not prevent 10–20% of oocytes from maturing. Therefore, other unknown oocyte meiosis-related factors may be regulated by HDAC3 in GCs.

The reasons why altered expression of more genes was observed following treatment with the HDACi 4b inhibitor compared with genetic loss of HDAC3 could be versatile. According to our study, the expression of *Hdac3* in the ovarian GCs was only partially depleted whenever *Hdac3* was deleted in *Hdac3*^cKO^ females by administration of tamoxifen because of the low efficiency of induced gene knockout. As one of the inhibitors of HDAC3, HDACi 4b is also functional in inhibiting the activity of HDAC1. However, HDACi 4b showed a ~3-, ~25-, and ~72-fold selectivity for HDAC3 over HDAC1, 2, and 8 respectively, and essentially no activity against other HDAC subfamily members^18^. The concentration of HDACi 4b we used in this paper was the lowest effective concentration based on our pre-assays, which means that HDACi 4b at this specific concentration could mimic the effect of HDAC3 depletion on AREG expression and oocyte maturation in vivo. In this study, more altered expression of genes was observed following HDACi 4b treatment, instead of the in vivo group (Supplementary Fig. S6). Taking into account these above reasons, it is therefore understandable the differences between the data obtained in vivo resulting from HDAC3 partially depleted GCs and the data obtained in vitro resulting from HDAC3 fully inhibited GCs. Anyway, the expression of *Areg* in ovarian GCs was significantly increased in both *Hdac3*^cKO^ mice and HDACi 4b-treated GCs, which is in agreement with other results obtained in vitro and in vivo. In sum, *Areg* is an essential target gene of HDAC3 in mediating gonadotropin action on oocyte maturation.

Although HDACi 4b preferentially targets HDAC3 and had limited in vitro and in vivo toxicity^18^, an evaluation of the safety of HDACi 4b is still necessary before blastocysts produced with this system could be considered for practical use. Despite that HDAC1/3 belongs to the same class and directly interact in some cases, their cell type- and developmental stage-specific physiological actions may be distinct^33^. Interestingly, among all class I family HDACs currently identified in humans^34^, only HDAC3 responded to LH in preovulatory follicles in this study.

Physiologically, ovarian GCs mediates the action of gonadotropins on oocyte maturation and ovulation in the periovulatory follicles. Our results proved that, although the transcription factors (such as SP1) of AREG exists before LH surge in mGCs, these transcription factors cannot initiate AREG expression because the accessibility of *Areg* promoter is blocked by HDAC3. The findings emphasized the pivotal role of epigenetic modification of histone acetylation for the specifically open and closed of FSH and LH target genes in mGCs during oocyte maturation.

## Methods

### Animal strains

C57BL/6J female mice were raised in colonies maintained by the investigators. Mice aged 21–23 days received an i.p. injection of 5 IU of PMSG (Sansheng Pharmaceutical Co., Ltd., Ningbo, China) to stimulate follicle growth. Forty-six to 48 h later, the mice received an i.p. injection of 5 IU of hCG (Sansheng Pharmaceutical Co., Ltd., Ningbo, China) to induce oocyte maturation and ovulation. *Hdac3*^*flox/flox*^ mice were purchased from the Jackson Laboratory (ME, USA), and *Foxl2*-*Cre*ER^T2^ mice were a gift from Dr. Liu Kui. All of these mice were housed under controlled temperature (22 °C) and light conditions (14 h light, 10 h darkness; lights on at 07:00 a.m.) and allowed free access to chow and water. Briefly, to obtain the *Hdac3*^cKO^ mice, we crossed the *Hdac3*^*flox/flox*^ females with *Hdac3*^*flox/flox*^ plus *Foxl2-Cre*ER^T2^ males without tamoxifen treatment. Therefore, the offsprings with *Hdac3*^*flox/flox*^ but without *Foxl2-Cre*ER^T2^ were used as controls. The experiments were performed in accordance with the principles and guidelines for the use of laboratory animals of China Agricultural University and approved by the Institutional Animal Care and Use Committee of China Agricultural University.

### Tamoxifen administration in *Foxl2*-*Cre*ER^T2^; *Hdac3*^*fl/fl*^ mice

Tamoxifen was resuspended to a concentration of 100 mg/mL in 95% ethanol and further diluted with corn oil to a final concentration of 20 mg/mL. A single i.p. injection of tamoxifen (20 mg/kg body weight) was administered, respectively, to 18-day-old *Hdac3*^*flox/flox*^; *Foxl2*-*Cre*ER^T2^ female mice and *Hdac3*^*flox/flox*^ female mice (controls).

### Chemicals, hormones, and media

All chemicals were purchased from Sigma-Aldrich (St. Louis, MO, USA) unless otherwise noted. Media used for all experiments were obtained from Gibco (Life Technologies, CA, USA). All of the antibodies against histone acetylation were purchased from Active Motif (Shanghai, China) with the exception of the H3K14ac antibody, which was purchased from EpiGentek (Farmingdale, NY, USA). Other antibodies used in this paper are presented in Supplementary Table 3.

### Follicle, COC, and mGC culture

Follicles were isolated from the ovaries of PMSG-primed mice and cultured in M199 medium with ITS (insulin–transferrin–sodium selenite media supplement). To investigate the effect of the HDAC3 inhibitor HDACi 4b (sc207902, Santa Cruz Biotechnology, CA, USA) on oocyte maturation in vitro, the follicles were cultured for 16 h with or without 5 μM HDACi 4b. Furthermore, to investigate the role of SP1 in AREG expression, 1 μM MIT, an SP1 inhibitor, was added to both HDACi 4b-treated follicles and LH-treated follicles for 4 h. Gene expression in all these groups was examined at the end of the culture period.

Previous studies reported that PMSG was able to mimic the action of FSH in follicle development, and hCG mimicked the effects of LH on oocyte maturation and ovulation^35–37^. Therefore, PMSG and hCG are widely used as substitutes for FSH and LH in others’ and our studies. Here COCs and mGCs were collected with a micropipette by puncturing large antral follicles with oocytes at the GV stage from the ovaries of mice that were pretreated with PMSG for 46–48 h. The collected mGCs and COCs were maintained in hypoxanthine (HX)-IVM medium (M199 medium containing 4 mM HX, 75 g/mL penicillin G, 50 g/mL streptomycin sulfate, 0.29 mM pyruvate, and 3 mg/mL bovine serum albumin (BSA)). To promote cell attachment, mGCs were cultured in IVM medium with 10% fetal bovine serum (FBS). Before COC culture, mGCs were treated with or without 5 μM HDACi 4b overnight to investigate the effect of mGC-derived HDAC3 on oocyte maturation. Then COCs were added to the mGC feeders and cocultured for 16 h with 30 nM NPPC. Furthermore, to elucidate whether AREG mediates the effects of HDAC3 on oocyte maturation, 1 μM AG1478, an EGFR inhibitor, was added to the mGC feeder medium. The GVBD ratio of oocytes in these COCs was analyzed. In addition, samples of mGCs and COCs were centrifuged, frozen, and stored at −80 °C for subsequent mRNA or protein expression analysis.

Follicles, oocytes, COCs, and mGCs were cultured at 37 °C in a controlled atmosphere of 5% CO_2_ and 95% air throughout the study.

### RNA preparation and analysis

RNA was isolated from cultured cells and follicles using TRIzol Reagent (Invitrogen, Life Technologies, CA, USA) according to the manufacturer’s protocol. The quantity and quality of total RNA were assessed using a Nanodrop spectrophotometer (Thermo Scientific, USA). Reverse transcription (RT) (Promega Reverse Transcription System) was performed using 1 μg of total RNA per sample. qPCR was conducted and analyzed on an ABI 7500 Sequence Detection System (Applied Biosystems, USA) using a standard protocol. The primers used for test genes are listed in Supplementary Table 1. For RNA-seq analysis of ovarian GCs from *Hdac3*^*flox/flox*^ and *Hdac3*^cKO^ mice, total RNA extracted from *Hdac3*^*flox/flox*^ and *Hdac3*^cKO^ mouse ovaries was analyzed by Annoroad Gene Technology Co., Ltd. (Beijing, PR China). Furthermore, for single-cell RNA-seq analysis of oocytes cultured with or without HDACi 4b, RNA was extracted from 35 oocytes per group, and transcriptome analysis was performed by Annoroad Gene Technology Co., Ltd. (Beijing, PR China).

### Chromatin immunoprecipitation

To clarify whether HDAC3 directly binds to the *Areg* promoter, ChIP analysis was performed as described in the ChIP Assay Kit instructions (17–371, Millipore, MA, USA). In brief, cells extracted from mouse ovaries in vivo or cultured cells in vitro were cross-linked with 1% formaldehyde for 10 min at room temperature and neutralized by glycine at a final concentration of 0.125 M. After three washes with ice-cold phosphate-buffered saline (PBS), cell pellets were lysed in 1% sodium dodecyl sulfate (SDS) buffer containing a protease inhibitor. Then chromatin was sheared by sonication until the average DNA length was approximately 300–500 bp, as evaluated by 1% agarose gel electrophoresis. Sheared chromatin was diluted in ChIP dilution buffer to a final SDS concentration of 0.1%. Then salmon sperm DNA/protein agarose slurry was added to preclear the chromatin solution. One percent of the chromatin fragment sample was stored at 4 °C for later use as nonprecipitated total chromatin (input) for normalization. The remaining chromatin fragments (99%) were incubated overnight at 4 °C with 3 μg of anti-HDAC3 antibody. Normal rabbit IgG (17–601, Millipore, MA, USA) was used as a negative control for nonspecific immunoprecipitation. The chromatin–antibody complex was incubated with protein A/G beads for 1 h at 4 °C. The antibody/DNA complexes on agarose beads were collected by centrifugation. The beads were washed with various buffers in the following order: low-salt immune complex buffer, high-salt immune complex buffer, LiCl immune complex buffer, and TE buffer provided by the supplier. Then the beads were suspended in elution buffer, and precipitated protein/DNA complexes were eluted from the antibodies/beads. The resulting protein/DNA complexes were then subjected to cross-link reversal in 0.2 M NaCl at 65 °C for 10 h, followed by the addition of 10 mM EDTA, 40 mM Tris-HCl, and 40 μg/ml proteinase K and incubation at 55 °C for 2 h. DNA was purified by phenol/chloroform extraction and ethanol precipitation. Specific primers (Supplementary Table 2) were used to detect immunoprecipitated chromatin fragments and input chromatin.

### Immunofluorescence

Ovaries and follicles were fixed in cold 4% paraformaldehyde (PFA) for 48 and 24 h, respectively. Then the samples were dehydrated in ethanol and toluene, embedded in paraffin, and sectioned at 5-μm thickness. Ovaries and follicles were transferred to 3-aminopropyl-triethoxysilane-treated microscope slides (ZLI-9001, Zhongshan Company, Beijing, China) for immunofluorescence staining. In brief, sections were deparaffinized and rehydrated, and antigen retrieval was performed by microwaving for 15 min in 0.01% sodium citrate buffer (pH 6.0). For immunofluorescence analysis, sections were blocked with 10% normal donkey serum and incubated overnight at 4 °C with primary antibodies against HDAC3 before incubation with Alexa Fluor 488- or 555-conjugated secondary antibodies (1:100, Invitrogen, Life Technologies, CA, USA) for 1 h at 37 °C and propidium iodide (421301, BioLegend, CA, USA) or Hoechst 33342 (B2261; Sigma-Aldrich, USA) as a nuclear counterstain. Samples were observed under a microscope.

### Immunohistochemistry (IHC)

For IHC, ovary sections were treated with 3% H_2_O_2_ in PBS for 20 min to quench endogenous peroxidase activity. Nonspecific binding was blocked with 5% BSA in PBS. Sections were then incubated overnight at 4 °C with the primary antibodies, incubated for 20 min at room temperature with horseradish peroxidase-labeled goat anti-rabbit IgG (Zhongshan Company, Beijing, China), and rinsed with PBS. The antibody complex was detected using DAB reagent according to the manufacturer’s instructions (Zhongshan Company, Beijing, China).

### Periodic acid Schiff (PAS) staining

To detect the corpora amylacea marker PAS, the sections were incubated in 0.5% periodic acid (Solarbio Company, Beijing, China) for 5 min at room temperature, followed by washing in tap water for 1 min. Then the sections were incubated in Schiff reagent (Solarbio Company, Beijing, China) for 10 min, followed by washing in tap water for 10 min. After the sections were rinsed in PBS for 10 min, the nuclei were stained with hematoxylin.

### Coimmunoprecipitation

Fresh GCs were collected from the ovaries of 22–24-day-old female mice following stimulation with 5 IU of PMSG for 46 h. Lysis buffer was added, and samples were centrifuged to obtain total protein. HDAC3 immunoprecipitation was performed according to the protocols of Dynabeads Protein G Immunoprecipitation Kit (Invitrogen, USA), as summarized below. Dynabeads were resuspended in Binding & Washing buffer containing 5 μg of anti-HDAC3 antibody and incubated for 40 min with rotation at room temperature. Dynabead–antibody complexes were then washed with Binding and Washing buffer. Then protein lysates were added for 1 h at room temperature with rotation, and the Dynabead–antibody–antigen complexes were washed thrice. Next, the complex was gently resuspended in 20 μL of elution buffer and incubated for 5 min at 70 °C. The supernatant was transferred to a new tube and boiled in elution buffer for 10 min in a water bath at 100 °C. The nonspecific binding control was incubated with normal rabbit IgG (sc-2763, Santa Cruz Biotechnology, CA, USA). The supernatants of both the control and HDAC3 groups were subjected to liquid chromatography coupled with MS in the MS laboratory of China Agriculture University to identify all proteins that interacted with HDAC3.

### Immunoblotting

Total protein was extracted in WIP (CellChip, BJ Biotechnology Co., Ltd., Beijing, China) as recommended by the manufacturer. In brief, total protein from each sample was separated by 10% SDS-polyacrylamide gel electrophoresis and then electrophoretically transferred onto polyvinylidene fluoride membranes (IPVH00010, Millipore, MA, USA). The membranes were then incubated for 1 h at room temperature with 5% BSA, followed by an overnight incubation at 4 °C with an anti-HDAC3 antibody. After being washed in TBST, the membranes were incubated with secondary antibody (1:5000, ZB-2301 and ZB-2305, ZSGB-BIO, Beijing, China) in TBST, followed by detection with the SuperSignal chemiluminescent detection system (34080, Thermo Scientific, CA, USA). Glyceraldehyde 3-phosphate dehydrogenase was used as an internal control.

### DNA pulldown assay and bioinformatic analysis with JASPAR

To identify transcription factors that bind to the *Areg* promoter, DNA pulldown assays were performed as described previously^38^. Briefly, fresh GCs were collected from the ovaries of 22–24-day-old female mice stimulated with 5 IU of PMSG for 46 h and the nuclei were extracted from GCs with Buffer A (10 mM HEPES, pH 7.9, 1.5 mM MgCl_2_, 10 mM KCl, 300 mM sucrose, and 0.5% NP-40). Nuclear protein was extracted from GC nucleus with NE (20 mM HEPES (pH7.9), 1.5 mM MgCl2, 0.5 M NaCl, 0.2 mM EDTA and 20% glycerol) and NET (Make up to 1% Triton-X-100, 1 mM dithiothreitol (DTT), 1 mM phenylmethylsulfonyl fluoride in NE buffer) buffer as described^38^. Then primers targeting the *Areg* promoter (−343 to −150) were synthesized with a 5′ biotin label, resulting in 5′-biotinylated double-strand DNA probes. The DNA probes were incubated overnight with the extracted nuclear proteins. The DNA–protein complexes were incubated with streptavidin-agarose beads (20347, Thermo Scientific, CA, USA) for 1 h. The DNA–protein–streptavidin bead complexes were washed three times with PBS. Finally, the complexes were eluted and subjected to MS analysis in the MS laboratory of China Agriculture University. For the bioinformatic analysis of potential transcription factor binding to the *Areg* promoter with JASPAR, we uploaded the *Areg* promoter sequence (from −343 to −150) onto the http://jaspar.genereg.net/ website.

### Cross-linking Co-IP and reverse cross-linking Co-IP

To identify all the interactors of HDAC3 at chromatin, we performed cross-linking Co-IP assays as described previously^24^. Briefly, fresh GCs were collected from the ovaries of 22–24-day-old female mice stimulated with 5 IU of PMSG for 46 h. Nuclear proteins were extracted from GCs with Buffer A (10 mM HEPES, pH 7.9, 1.5 mM MgCl_2_, 10 mM KCl, 300 mM sucrose, and 0.5% NP-40) as described^39^. Then the nuclei were fixed with 1% formaldehyde diluted in PBS for 10 min at 37 °C. Cross-linked samples were quenched with glycine, washed once with PBS, and resuspended in 1 ml NCB (20 mM Tris-HCl pH 8, 100 mM KCl, 5 mM MgCl_2_, 10% glycerol, 0.1% NP-40, and 1 mM DTT) supplemented with EDTA-free protein inhibitor cocktail (Millipore). Lysates were sonicated with a probe sonicator (Ningbo, China). Then the cross-linked chromatin extracts were cleared by centrifugation and subsequently treated with 1000 Kunitz units of Benzonase (Sigma) for 30 min at 30 °C. The extracts were immunoprecipitated overnight with anti-HDAC3 magnetic beads (16–663, Millipore, MA, USA) or normal IgG magnetic beads. Then the complexes were washed three times and eluted with elution buffer for MS analysis in the MS laboratory of China Agriculture University. To confirm the interaction between FOXO1 and HDAC3, the cross-linking of the proteins eluted from the complexes was reversed by heating at 65 °C overnight with 0.2 M NaCl. Then the reverse cross-linked proteins were analyzed by western blotting.

### Oocyte IVM, IVF, and early embryo IVC

Four- to 5-week-old mice were used for this experiment. The mGCs retrieved from mice were cultured in Nunc IVF 4-well dishes (Thermo Scientific, Life Technologies, CA, USA) with IVM medium containing 10% FBS with or without 5 μM HDAC3 inhibitor. The medium was covered with mineral oil (M8410, Sigma). The collected COCs were cocultured with mGC feeders. COCs in both groups were digested with hyaluronidase (90017, Vitrolife, Sweden) before examining meiosis progression. Oocytes at the MII stage in both groups were used for IVF. Mouse sperm capacitation was induced with IVF medium (Vitrolife, Sweden) for 1 h. Then sperm were mixed with MII oocytes in IVF medium for 6 h. Fertilization was confirmed by monitoring pronuclear formation. Then zygotes were transferred to G1 plus medium (Vitrolife, Sweden) and cultured until the embryos reached the eight-cell stage. Then zygotes were transferred into G2 plus medium (Vitrolife, Sweden) for further culture. The number of blastocysts was calculated and analyzed. The embryos were transferred into the uterus of a 2.5-day postcoital pseudopregnant female mice as previously described^38^.

### TUNEL

For TUNEL assays, ovaries were collected from *Hdac3*^*flox/flox*^ and *Hdac3*^cKO^ mice and fixed in cold 4% PFA for 1 h. The tissues were embedded in paraffin wax and serially sectioned at 5 μm. Apoptotic cells were detected using a TUNEL Apoptosis Detection Kit (Invitrogen, Life Technologies, USA) after HDAC3 immunostaining.

### Statistical analysis

All analysis were performed using Graph Pad Prism version 7 for Windows (GraphPad Software, La Jolla, CA, USA). Data for RT-PCR assays, follicle and oocyte counting experiments, and breeding tests are presented as mean ± SEM, and each experiment was performed in triplicate. Data were analyzed using *t* test or analysis of variance. Values of *P* < 0.05 were considered statistically significant.

### Reporting summary

Further information on research design is available in the Nature Research Reporting Summary linked to this article.

## Supplementary information

Supplementary information

Description of Additional Supplementary Files

Supplementary Data 1

Supplementary Data 2

Supplementary Data 3

Supplementary Data 4

Supplementary Data5

Supplementary Data 6

Supplementary Data 7

Supplementary Data 8

Reporting Summary

## Data Availability

RNA-seq data have been submitted to the NCBI Gene Expression Omnibus (GEO; http://www.ncbi.nlm.nih.gov/geo/) under accession number GSE139666 for the SuperSeries record of the three groups of RNA-seq data, including GSE139663, GSE139664, and GSE139665, respectively, for the transcriptome of *HDAC3*^cKO^ mice ovarian mGCs, the cultured ovarian mGCs treated with HDACi 4b, and the cultured oocytes treated with HDACi 4b. Proteomics data of Fig. 4 are available in Supplementary Data 4–8. The reporting summary and editorial checklist for this article are available as a Supplementary file.

## Source Data

Source Data

## References

[1] Hsieh M (2007). Luteinizing hormone-dependent activation of the epidermal growth factor network is essential for ovulation. Mol. Cell. Biol..

[2] Conti M, Hsieh M, Zamah AM, Oh JS (2012). Novel signaling mechanisms in the ovary during oocyte maturation and ovulation. Mol. Cell. Endocrinol..

[3] Zhang M, Su YQ, Sugiura K, Xia G, Eppig JJ (2010). Granulosa cell ligand NPPC and its receptor NPR2 maintain meiotic arrest in mouse oocytes. Science.

[4] Richard FJ, Tsafriri A, Conti M (2001). Role of phosphodiesterase type 3A in rat oocyte maturation. Biol. Reprod..

[5] Vaccari S, Weeks JL, Hsieh M, Menniti FS, Conti M (2009). Cyclic GMP signaling is involved in the luteinizing hormone-dependent meiotic maturation of mouse oocytes. Biol. Reprod..

[6] Park JY (2004). EGF-like growth factors as mediators of LH action in the ovulatory follicle. Science.

[7] Wang X (2018). High level of C-type natriuretic peptide induced by hyperandrogen-mediated anovulation in polycystic ovary syndrome mice. Clin. Sci..

[8] Liu W (2017). Estrogen receptors in granulosa cells govern meiotic resumption of pre-ovulatory oocytes in mammals. Cell Death Dis..

[9] Zhang M, Xia G (2012). Hormonal control of mammalian oocyte meiosis at diplotene stage. Cell. Mol. Life Sci..

[10] Fang J (2016). Involvement of protein acyltransferase ZDHHC3 in maintaining oocyte meiotic arrest in *Xenopus laevis*. Biol. Reprod..

[11] Yoshida N, Brahmajosyula M, Shoji S, Amanai M, Perry AC (2007). Epigenetic discrimination by mouse metaphase II oocytes mediates asymmetric chromatin remodeling independently of meiotic exit. Dev. Biol..

[12] Lee L (2013). Changes in histone modification and DNA methylation of the StAR and Cyp19a1 promoter regions in granulosa cells undergoing luteinization during ovulation in rats. Endocrinology.

[13] Strahl BD, Allis CD (2000). The language of covalent histone modifications. Nature.

[14] Qu F (2012). A molecular mechanism underlying ovarian dysfunction of polycystic ovary syndrome: hyperandrogenism induces epigenetic alterations in the granulosa cells. J. Mol. Med..

[15] Grunstein M (1997). Histone acetylation in chromatin structure and transcription. Nature.

[16] Alenghat T (2013). Histone deacetylase 3 coordinates commensal-bacteria-dependent intestinal homeostasis. Nature.

[17] Zheng W (2014). Two classes of ovarian primordial follicles exhibit distinct developmental dynamics and physiological functions. Hum. Mol. Genet..

[18] Jia H (2012). Histone deacetylase (HDAC) inhibitors targeting HDAC3 and HDAC1 ameliorate polyglutamine-elicited phenotypes in model systems of Huntington’s disease. Neurobiol. Dis..

[19] Hernandez-Gonzalez I (2006). Gene expression profiles of cumulus cell oocyte complexes during ovulation reveal cumulus cells express neuronal and immune-related genes: does this expand their role in the ovulation process?. Mol. Endocrinol..

[20] Wang Y (2013). Epidermal growth factor receptor signaling-dependent calcium elevation in cumulus cells is required for NPR2 inhibition and meiotic resumption in mouse oocytes. Endocrinology.

[21] Karagianni P, Wong J (2007). HDAC3: taking the SMRT-N-CoRrect road to repression. Oncogene.

[22] Guenther MG, Barak O, Lazar MA (2001). The SMRT and N-CoR corepressors are activating cofactors for histone deacetylase 3. Mol. Cell. Biol..

[23] Watson PJ, Fairall L, Santos GM, Schwabe JW (2012). Structure of HDAC3 bound to co-repressor and inositol tetraphosphate. Nature.

[24] Armour SM (2017). An HDAC3-PROX1 corepressor module acts on HNF4alpha to control hepatic triglycerides. Nat. Commun..

[25] Bonnet A, Dalbiès-Tran R, Sirard MA (2008). Opportunities and challenges in applying genomics to the study of oogenesis and folliculogenesis in farm animals. Reproduction.

[26] Zamah AM (2010). Human oocyte maturation is dependent on LH-stimulated accumulation of the epidermal growth factor-like growth factor, amphiregulin. Hum. Reprod..

[27] Conti M (2002). Role of cyclic nucleotide signaling in oocyte maturation. Mol. Cell. Endocrinol..

[28] Jurema MW, Nogueira D (2006). In vitro maturation of human oocytes for assisted reproduction. Fertil. Steril..

[29] Feng R (2016). Mutations in TUBB8 and human oocyte meiotic arrest. N. Engl. J. Med..

[30] Swain JE, Pool TB (2008). ART failure: oocyte contributions to unsuccessful fertilization. Hum. Reprod. Update.

[31] Chen J (2013). Somatic cells regulate maternal mRNA translation and developmental competence of mouse oocytes. Nat. Cell Biol..

[32] Santos MA, Kuijk EW, Macklon NS (2010). The impact of ovarian stimulation for IVF on the developing embryo. Reproduction.

[33] Thomas EA (2008). The HDAC inhibitor 4b ameliorates the disease phenotype and transcriptional abnormalities in Huntington’s disease transgenic mice. Proc. Natl Acad. Sci. USA.

[34] Xu WS, Parmigiani RB, Marks PA (2007). Histone deacetylase inhibitors: molecular mechanisms of action. Oncogene.

[35] Popova E, Krivokharchenko A, Ganten D, Bader M (2002). Comparison between PMSG- and FSH-induced superovulation for the generation of transgenic rats. Mol. Reprod. Dev..

[36] Pramoda S, Saidapur SK (1984). Preponement of ovarian follicular development in the frog, *Rana tigerina*, using PMSG, HCG, growth hormone, heteroplastic pituitary pars distalis homogenate, FSH and LH. Indian J. Exp. Biol..

[37] Neal P, Baker TG (1974). Response of mouse ovaries in vivo and in organ culture to pregnant mare’s serum gonadotrophin and human chorionic gonadotrophin. 3. Effect of age. J. Reprod. Fertil..

[38] Singer O, Tiscornia G, Ikawa M, Verma IM (2006). Rapid generation of knockdown transgenic mice by silencing lentiviral vectors. Nat. Protoc..

[39] Wu, K. K. in *Gene Mapping, Discovery, and Expression: Methods and Protocols* (ed. Bina, M.) 281–290 (Humana Press, Totowa, NJ, 2006).

