# Supplementary material for: HDAC3 maintains oocyte meiosis arrest by repressing amphiregulin expression before the LH surge
Source: Nat Commun. 2019 Dec 16;10:5719. doi: 10.1038/s41467-019-13671-8 (PMC6915726; doi:10.1038/s41467-019-13671-8)
Supplement: Supplementary file 1 — Supplementary information [file 41467_2019_13671_MOESM1_ESM.pdf]

Supplementary Information

HDAC3 maintains oocyte meiosis arrest by repressing amphiregulin expression before the LH surge by Wang et al.

Supplementary Figures

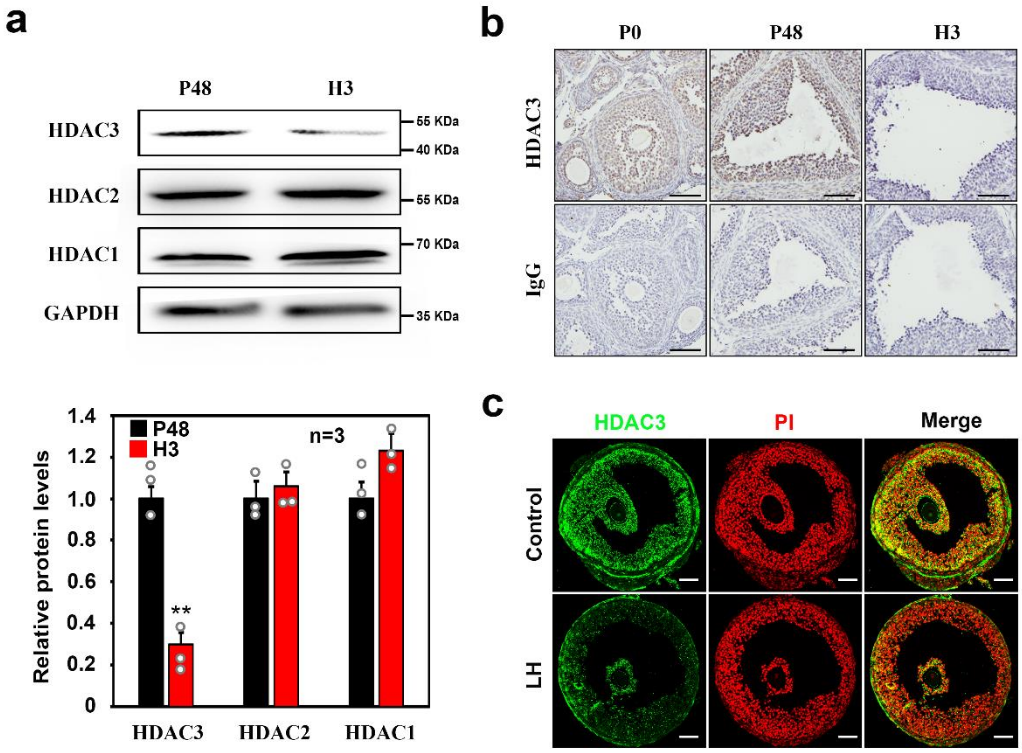

Supplementary Figure 1. LH decreases HDAC3 expression in ovarian GCs during oocyte maturation. **a** Western blot analysis revealed that among the class I family of HDACs, HDAC3 was specifically decreased by LH in GCs *in vivo*. n = 3 biologically independent experiments. Data are presented as mean ± SEM, Asterisks (*) indicate significant differences at ***P* = 0.004 determined by the two-side *t*-test. **b** HDAC3 immunostaining revealed that LH decreased HDAC3 levels *in vivo*. Scale bar=100 μm. n = 3 biologically independent experiments. **c** LH decreased HDAC3 levels in follicles cultured *in vitro*. HDAC3 in green; nuclear DNA in red (propidium iodide, PI) indicates. Scale bar=100 μm. n = 3 biologically independent experiments. Source data are provided as a Source Data file.

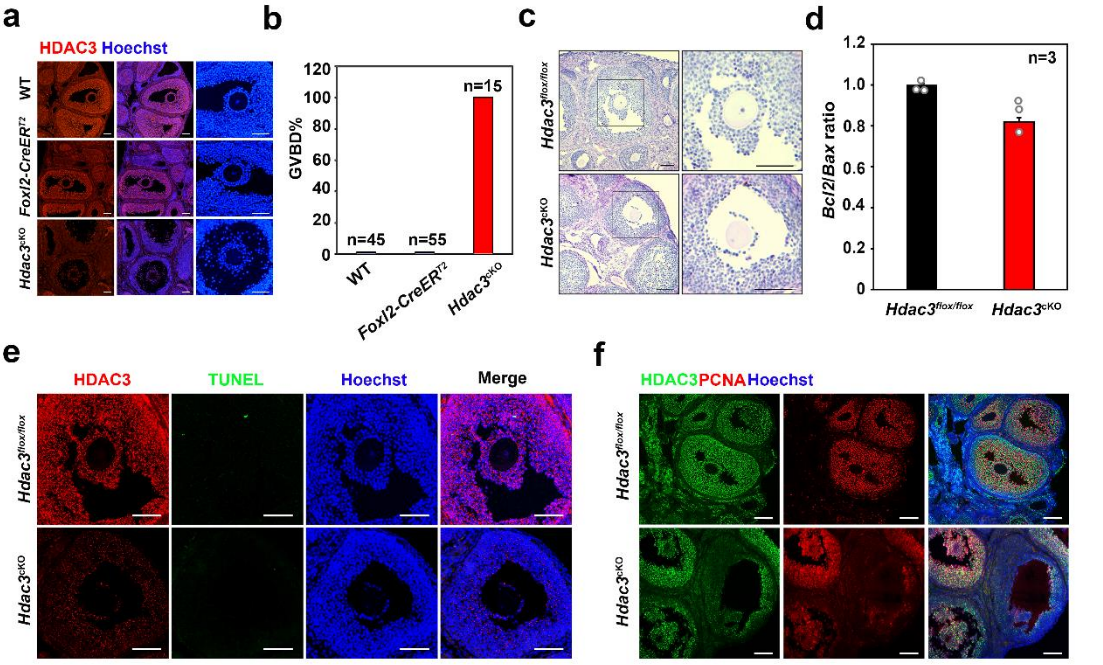

Supplementary Figure 2. *Hdac3* knockout in ovarian GCs mimics LH action on oocyte maturation and GCs proliferation. **a** HDAC3 (red) immunostaining in the different mouse models revealed that unlike the initiated oocyte meiosis resumption in $Hdac3^{flox/flox}$; *Foxl2-Cre*ER^{T2} female mice, the oocytes in wild type (WT) mice and $Foxl2\text{-}CreER^{T2}$ mice were both arrested at GV stage. Scale bar = 100 µm. Nuclear DNA in indicated in blue (Hoechst). **b** Oocyte maturation (GVBD) rates in follicles with or without HDAC3 expression in the different mouse genotypes. **c** The oocytes within follicles at either GV stage in $Hdac3^{flox/flox}$ or GVBD stage in $Hdac3^{cKO}$ ovaries. Cytoplasm were shown by periodic acid schiff (PAS) staining. Nuclei were dyed with hematoxylin. Scale bar=100 µm. n = 3 biologically independent experiments. **d** Quantitative real-time PCR analysis of the *Bcl2*/*Bax* ratio in $Hdac3^{flox/flox}$ and $Hdac3^{cKO}$ GCs. **e** Representative micrographs showing TUNEL staining (green) in $Hdac3^{flox/flox}$ and $Hdac3^{cKO}$ GCs. HDAC3 in red and nuclear in blue (Hoechst), scale bar=40 µm. n = 3 biologically independent experiments. **f** Immunostaining of PCNA (red) revealed that the HDAC3 (green) conditional knockout in GCs resulted in the termination of GC proliferation. Nuclei were dyedwith Hoechst (blue), scale bar=100 µm. n = 3 biologically independent experiments. Source data are provided as a Source Data file.

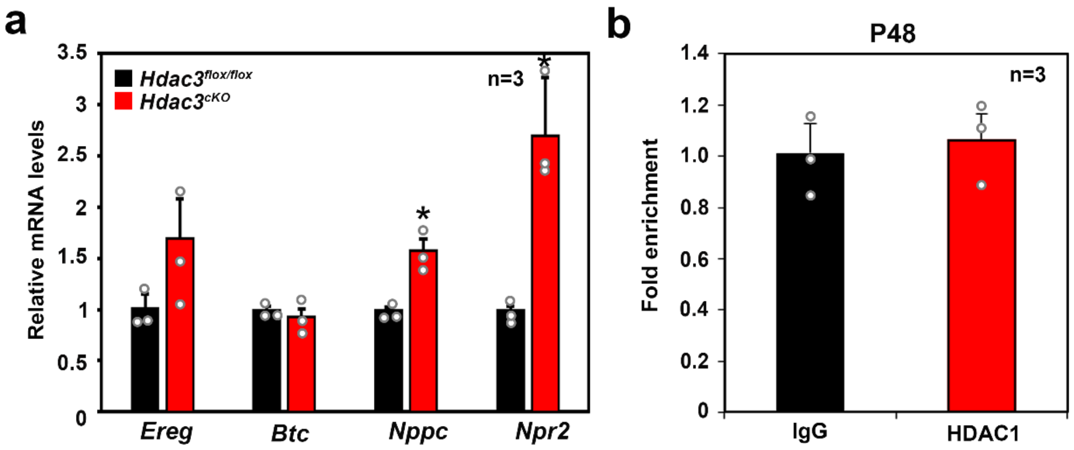

Supplementary Figure 3. HDAC3 rather than HDAC1 repressed *Areg* expression. **a** The mRNA levels of oocyte maturation-related genes of *Ereg*, *Btc*, *Nppc* and *Npr2* in *Hdac3*$^{flox/flox}$ mice and *Hdac3*^{cKO} mice. **b** A ChIP-qPCR revealed that HDAC1 was unable to bind to the *Areg* promoter (the region from -343 to -150) before the LH surge. Data are presented as mean ± SEM, Asterisks (*****) indicate significant differences at *****$P < 0.05$ determined by the two-side *t*-test. Source data are provided as a Source Data file.

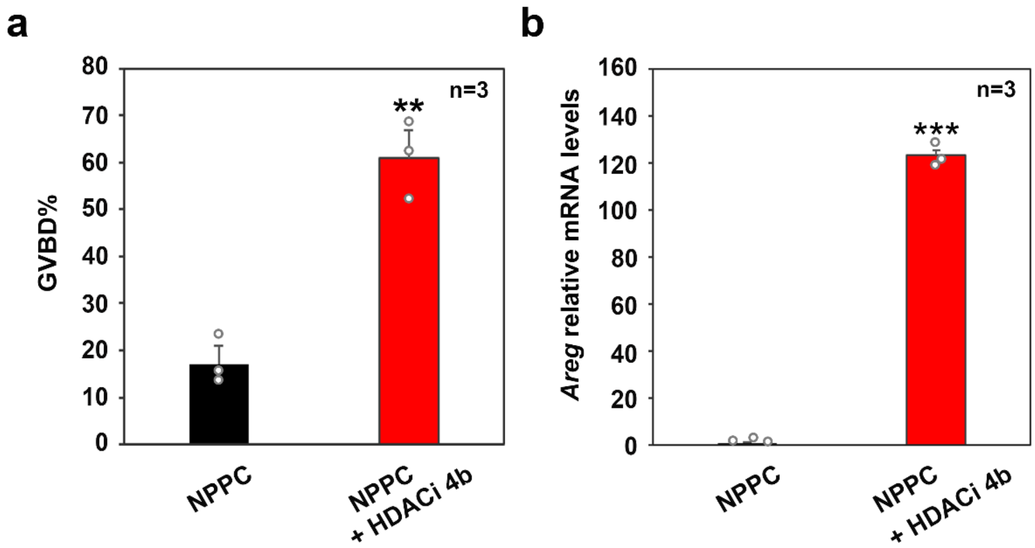

Supplementary Figure 4. HDACi 4b promoted the oocyte maturation in cultured COCs via inducing *Areg* expression in cumulus cells in NPPC-M199 medium. **a** Oocyte maturation rate (indicated by GVBD ratio) was significantly improved by HDACi 4b. HDACi 4b, the HDAC3 inhibitor. **b** The mRNA level of *Areg* in response to HDACi 4b was examined by quantitative real-time PCR analysis in our culture system. Data are presented as mean ± SEM, Asterisks (*) indicate significant differences at ***P* = 0.003 and ****P* = 5.05E-6 determined by the two-side *t*-test. Source data are provided as a Source Data file.

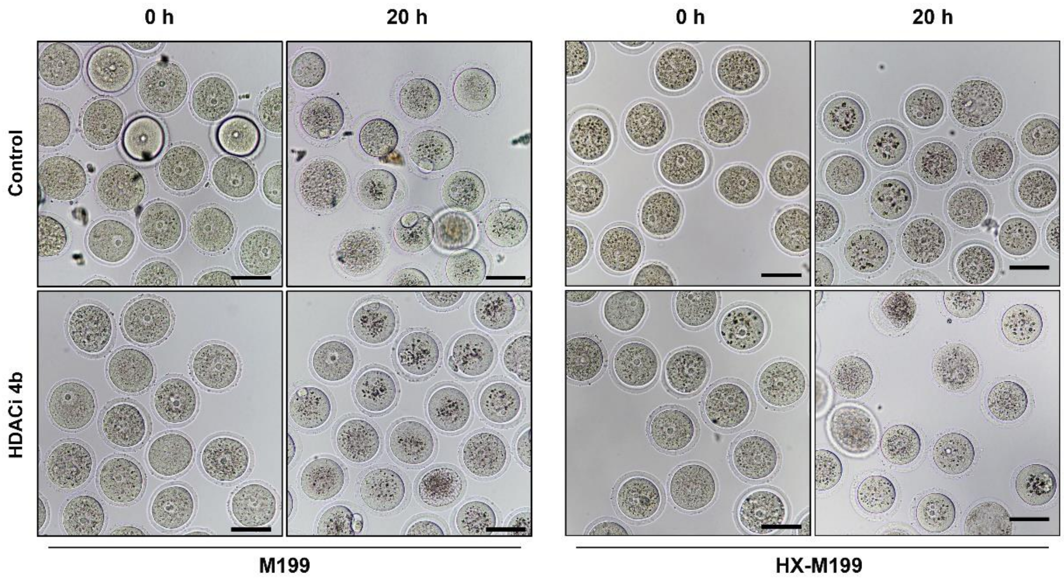

Supplementary Figure 5. HDACi 4b had no direct effect on the nude oocyte meiotic maturation. Nude oocytes recovered from wild type females were respectively cultured in HX-M199 or M199 medium with or without HDACi 4b for 20 h before examining the maturation rate. HDACi 4b, the HDAC3 inhibitor. Scale bar=100 μm. n = 3 biologically independent experiments.

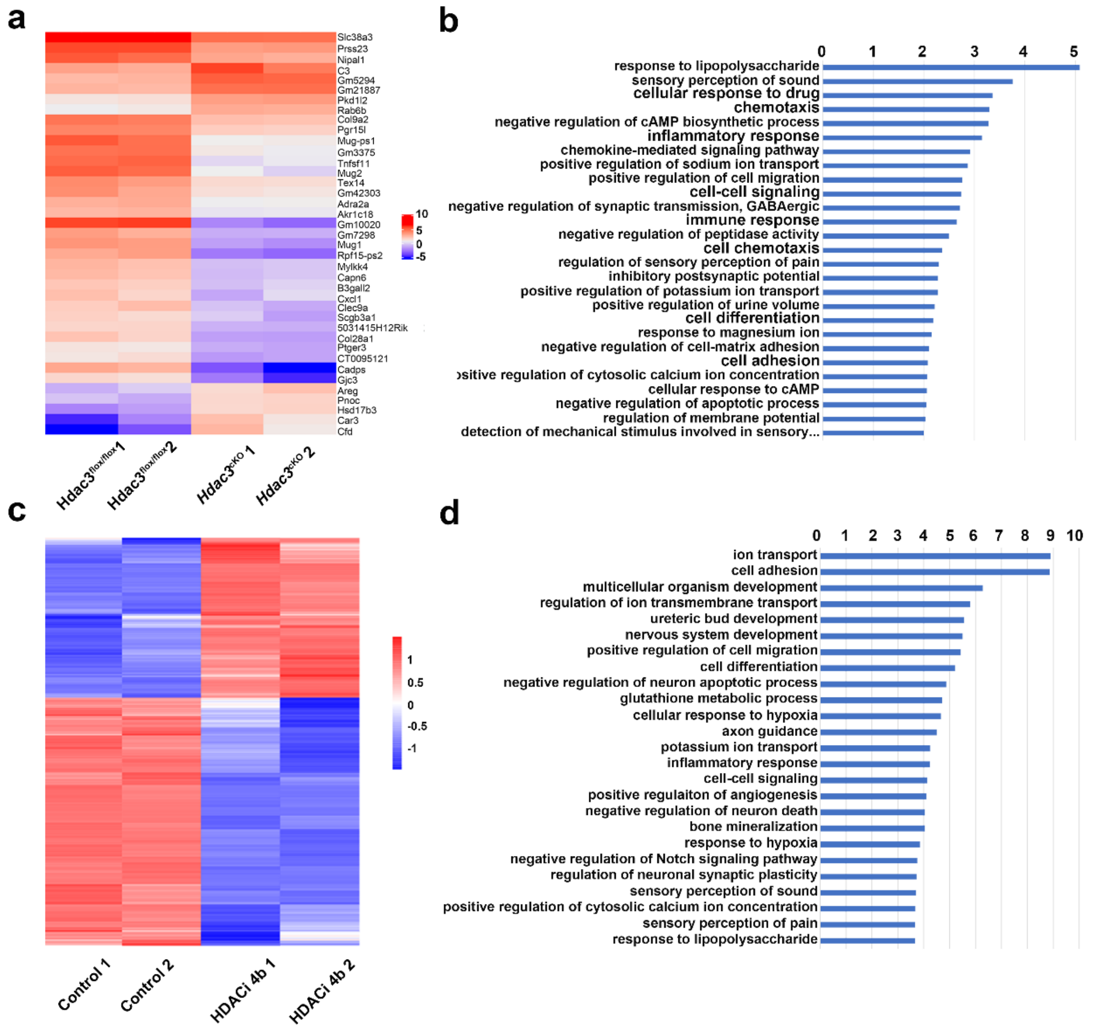

Supplementary Figure 6. RNAseq analysis for the GCs of HDAC3 depletion *in vivo* and inhibition *in vitro*. **a** Heatmap illustrated the GCs expressed genes with the most pronounced changes between *Hdac3*$^{flox/flox}$ and *Hdac3*^{cKO} mice. **b** Gene ontology (GO) analysis of the differently expressed genes between *Hdac3*$^{flox/flox}$ and *Hdac3*^{cKO} mice. **c** Heatmap illustrated the GCs expressed genes with the most pronounced changes between Control and HDAC3 inhibited group *in vitro*. **d** Gene ontology (GO) analysis of the differently expressed genes between Control and HDAC3 inhibited group *in vitro*.

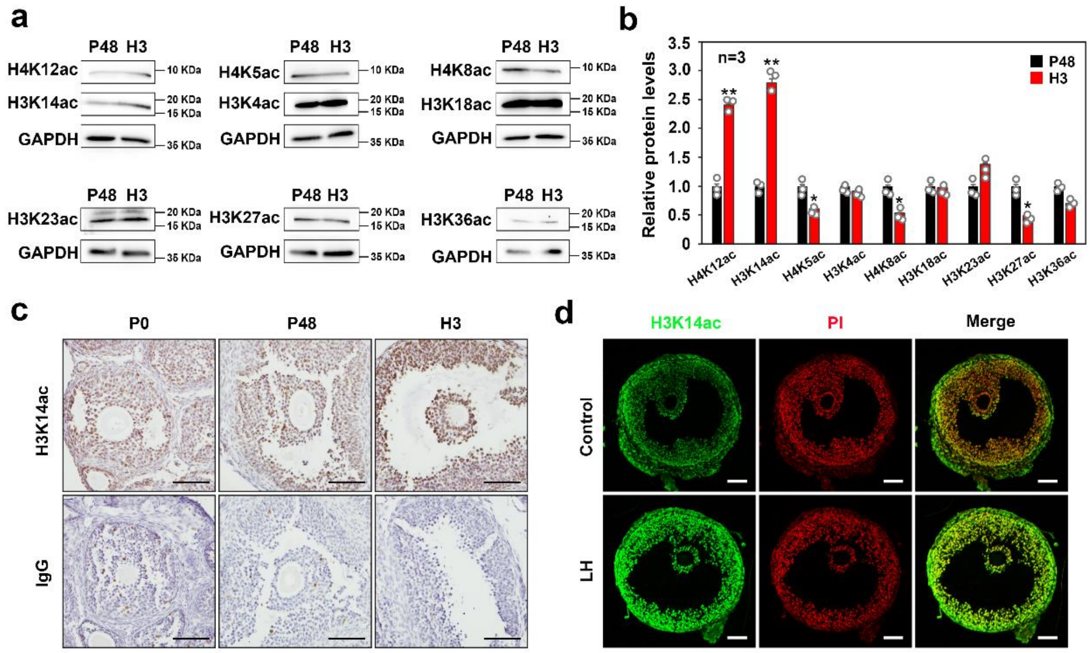

Supplementary Figure 7. LH induced the acetylation of H3K14 *in vivo* and *in vitro*. **a, b** Western blot analysis revealed the effect of LH on the acetylation of histones in GCs *in vivo*. n = 3 biologically independent experiments. Data are presented as mean ± SEM, Asterisks (*****) indicate significant differences at *****$P < 0.05$ and ******$P < 0.01$ determined by the two-side *t*-test. **c** H3K14ac immunostaining revealed that LH increased the acetylation levels of H3K14 *in vivo*. Scale bar=100 μm. n = 3 biologically independent experiments. **d** LH increased H3K14ac levels in follicles cultured *in vitro*. H3K14ac in green; nuclear DNA in red (PI) indicates. Scale bar=100 μm. n = 3 biologically independent experiments. Source data are provided as a Source Data file.

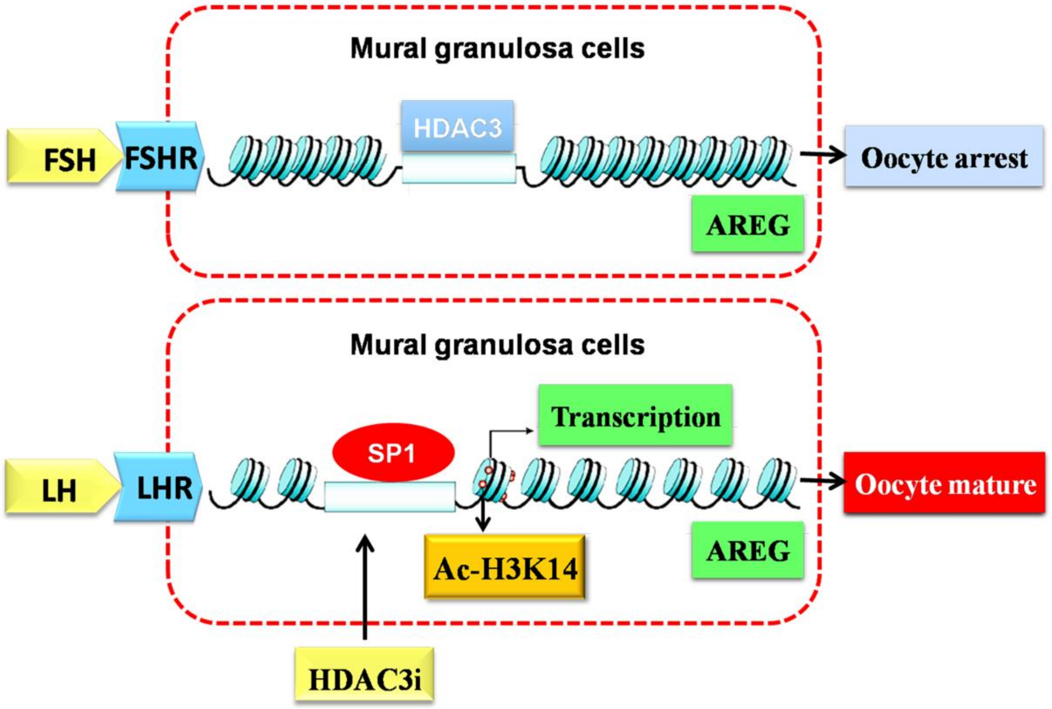

Supplementary Figure 8. The proposed signaling of HDAC3 in oocyte maturation.

Supplementary Table 1. List of primers used in quantitative real-time PCR.

Genes	Forwards (5' to 3')	Reverses (5' to 3')
Nppc	GGTCTGGGATGTTAGTGCAGCTA	TAAAAGCCACATTGCGTTGGA
Npr2	GCTGACCCGGCAAGTTCTGT	ACAATACTCGGTGACAATGCAGAT
Areg	GGTCTTAGGCTCAGGCCATTA	CGCTTATGGTGGAAACCTCTC
Btc	AAACCCACTTCTCTCGGTGC	AAACAGGTCCACTCGCTCAC
Ereg	GCATCCCAGGAGAATCCGAG	ACATCGCAGACCAGTGTAGC
Bcl2	GAACTGGGGGAGGATTGTGG	GCATGCTGGGGCCATATAGT
Bax	CTGGATCCAAGACCAGGGTG	GTGAGGACTCCAGCCACAAA
β-actin	CTTTGCAGCTCCTTCGTTGC	CCTTCTGACCCATTCCCACC

Supplementary Table 2. List of primers used in ChIP PCR.

Genes	Forwards (5' to 3')	Reverses (5' to 3')
Areg	GCTAACATCACTAAGCCACAG	CGAGCCCTGATTACGC

Supplementary Table 3. List of Primary antibodies used in immune detection.

Antibody	Catalog Code	Source	Host	Dilution		
				ChIP	IF/IHC	WB
HDAC3	Ab3070	Abcam	Rabbit	4 µg	1:1000	1:10000
HDAC1	17-608	Millipore	Mouse	4 µg		1:2000
HDAC2	AH382	Beyotime	Mouse			1:500
H3K14ac	A-4023	EPIGENTEK	Rabbit	4 µg	1:500	1:1000
PCNA	SC25280	Santa Cruz	Mouse		1:100	
AREG	SC74501	Santa Cruz	Mouse			1:500
FOXO1	2880	Cell signaling	Rabbit			1:1000
SP1	17-601	Millipore	Rabbit	4 µg		1:1000
GAPDH	G8795	Sigma-Aldrich	Mouse			1:5000
