# Supplementary material for: HDAC3 maintains oocyte meiosis arrest by repressing amphiregulin expression before the LH surge
Source: Nat Commun. 2019 Dec 16;10:5719. doi: 10.1038/s41467-019-13671-8 (PMC6915726; doi:10.1038/s41467-019-13671-8)
Supplement: Supplementary file 2 — Description of Additional Supplementary Files [file 41467_2019_13671_MOESM2_ESM.pdf]

Description of Additional Supplementary Files

File name: Supplementary Data 1
Description: Sequence analysis of transcriptome of denuded oocyte between control group and HDACi 4b-treated group.

File name: Supplementary Data 2
Description: Sequence analysis of transcriptome of ovarian follicles mural granulosa cells between *Hdac3*$^{flox/flox}$ mice and *Hdac3*^{cKO} mice.

File name: Supplementary Data 3
Description: Sequence analysis of transcriptome of cultured ovarian follicle mural granulosa cells between control group and HDACi 4b-treated group.

File name: Supplementary Data 4
Description: Proteomic analysis of HDAC3-binding proteins through co-immunoprecipitation coupled with mass spectrometry (LC-MS) with HDAC3 antibody.

File name: Supplementary Data 5
Description: Proteomic analysis of non-specific binding proteins through co-immunoprecipitation coupled with mass spectrometry (LC-MS) with IgG.

File name: Supplementary Data 6
Description: Proteomic analysis of HDAC3-binding proteins on chromatin through cross-linking co-immunoprecipitation coupled with mass spectrometry (LC-MS) with HDAC3 antibody and IgG.

File name: Supplementary Data 7
Description: Potential transcription factors that bind the *Areg* promoter obtained by JASPAR data.

File name: Supplementary Data 8
Description: Proteomic analysis of transcription factors that bind the *Areg* promoter through DNA-pulldown coupled with mass spectrometry (LC-MS).
