# Supplementary material for: HDAC3 maintains oocyte meiosis arrest by repressing amphiregulin expression before the LH surge
Source: Nat Commun. 2019 Dec 16;10:5719. doi: 10.1038/s41467-019-13671-8 (PMC6915726; doi:10.1038/s41467-019-13671-8)
Supplement: Supplementary file 11 — Reporting Summary [file 41467_2019_13671_MOESM11_ESM.pdf]

nature research

Corresponding author(s): Guoliang Xia

Last updated by author(s): Nov 3, 2019

Reporting Summary

Nature Research wishes to improve the reproducibility of the work that we publish. This form provides structure for consistency and transparency in reporting. For further information on Nature Research policies, see Authors & Referees and the Editorial Policy Checklist.

Statistics

For all statistical analyses, confirm that the following items are present in the figure legend, table legend, main text, or Methods section.

n/a	Confirmed	
☐	☒	The exact sample size (*n*) for each experimental group/condition, given as a discrete number and unit of measurement
☐	☒	A statement on whether measurements were taken from distinct samples or whether the same sample was measured repeatedly
☐	☒	The statistical test(s) used AND whether they are one- or two-sided<br>*Only common tests should be described solely by name; describe more complex techniques in the Methods section.*
☒	☐	A description of all covariates tested
☐	☒	A description of any assumptions or corrections, such as tests of normality and adjustment for multiple comparisons
☐	☒	A full description of the statistical parameters including central tendency (e.g. means) or other basic estimates (e.g. regression coefficient) AND variation (e.g. standard deviation) or associated estimates of uncertainty (e.g. confidence intervals)
☐	☒	For null hypothesis testing, the test statistic (e.g. *F*, *t*, *r*) with confidence intervals, effect sizes, degrees of freedom and *P* value noted<br>*Give P values as exact values whenever suitable.*
☒	☐	For Bayesian analysis, information on the choice of priors and Markov chain Monte Carlo settings
☐	☒	For hierarchical and complex designs, identification of the appropriate level for tests and full reporting of outcomes
☐	☒	Estimates of effect sizes (e.g. Cohen's *d*, Pearson's *r*), indicating how they were calculated

Our web collection on statistics for biologists contains articles on many of the points above.

Software and code

Policy information about availability of computer code

Data collection	No software was used.
Data analysis	All analysis were performed using Graph Pad Prism version 7 for Windows (GraphPad Software, La Jolla California USA).

For manuscripts utilizing custom algorithms or software that are central to the research but not yet described in published literature, software must be made available to editors/reviewers. We strongly encourage code deposition in a community repository (e.g. GitHub). See the Nature Research guidelines for submitting code & software for further information.

Data

Policy information about availability of data

All manuscripts must include a data availability statement. This statement should provide the following information, where applicable:

- Accession codes, unique identifiers, or web links for publicly available datasets
- A list of figures that have associated raw data
- A description of any restrictions on data availability

The source data of this study are provided as source data file. The accession codes of the RNA-seq are provided in the Data Available section.

Field-specific reporting

Please select the one below that is the best fit for your research. If you are not sure, read the appropriate sections before making your selection.

☒ Life sciences ☐ Behavioural & social sciences ☐ Ecological, evolutionary & environmental sciences

For a reference copy of the document with all sections, see nature.com/documents/nr-reporting-summary-flat.pdf

Life sciences study design

All studies must disclose on these points even when the disclosure is negative.

Sample size	For the analysis of HDAC3 conditional-knockout mice, ovaries from 3 wild-type (Hdac3flox/flox) female mice and 3 HDAC3 conditional KO female mice were collected to examine the oocyte meiosis progression and related gene expression. For follicles and COCs culture, 20 follicles and 50 COCs per group were used to examine the oocyte meiosis progression and related gene expression. For ovarian granulosa cell culture, the cells were collected from at least 3 female mice. For realtime-PCR, Western blotting, Co-IP and ChIP experiments, all of the samples were collected as described above. For the IVM and IVF of mice oocytes, 80 COCs per group were used to culture in vitro. As many as 10-15 blastocysts per group were transferred into surrogate mouse. According to the reports of others and ours (Meijia Zhang 2010), the sample sizes of each experiment were sufficient.
Data exclusions	No data were excluded from the analyses.
Replication	All of the experiments were repeated at least 3 times. And all of the results of each replication were consistent.
Randomization	All of the follicles, COCs, granulosa cells from mice were allocated into experimental groups randomly.
Blinding	The investigators were blinded to group allocation during data collection and/or analysis.

Reporting for specific materials, systems and methods

We require information from authors about some types of materials, experimental systems and methods used in many studies. Here, indicate whether each material, system or method listed is relevant to your study. If you are not sure if a list item applies to your research, read the appropriate section before selecting a response.

Materials & experimental systems

n/a	Involved in the study	
☐	☒	Antibodies
☒	☐	Eukaryotic cell lines
☒	☐	Palaeontology
☐	☒	Animals and other organisms
☒	☐	Human research participants
☒	☐	Clinical data

Methods

n/a	Involved in the study	
☒	☐	ChIP-seq
☒	☐	Flow cytometry
☒	☐	MRI-based neuroimaging

Antibodies

Antibodies used

Antibody	Catalog Code	Lot Code	Source	Host	Clone Name	Applicable
HDAC3	Ab3070	GR3202599-1	Abcam	Rabbit	Polyclonal	ChIP WB IF IP IHC
HDAC1	17-608	2504816	Millipore	Mouse	Monoclonal	WB ChIP
H3K14ac	A-4023	10905	EPIGENTEK	Rabbit	Polyclonal	ChIP WB IF IHC
HDAC2	AH382	121715160505	Beyotime	Mouse	Monoclonal	WB IP IHC
PCNA	SC25280	E0713	Santa Cruz	Mouse	Monoclonal	WB IF
SP1	17-601	2465224	Millipore	Rabbit	Polyclonal	WB ChIP
GAPDH	G8795		Sigma-Aldrich	Mouse	Monoclonal	WB
H3K4ac	39382	29108001	Active Motif	Rabbit	Polyclonal	ChIP WB IF
H3K18ac	39756	06710001	Active Motif	Rabbit	Polyclonal	ChIP WB IF
H3K23ac	39132	104	Active Motif	Rabbit	Polyclonal	ChIP WB IF
H3K36ac	39380	29108001	Active Motif	Rabbit	Polyclonal	ChIP WB IF
H4K5ac	39170	119	Active Motif	Rabbit	Polyclonal	ChIP WB IF
H4K8ac	61104	16111001	Active Motif	Rabbit	Polyclonal	ChIP WB IF
H4K12ac	39927		Active Motif	Rabbit	Polyclonal	ChIP WB IF
FOXO1	2880		Cell signaling	Rabbit	Monoclonal	ChIP WB IF
Secondary antibody:						
Anti-Rabbit	ZB2301		Zhongshan company	Goat	Polyclonal	WB
Anti-Mouse	ZB2305		Zhongshan Company	Goat	Polyclonal	WB
Anti-Rabbit	A21206		Invitrogen	Donkey	Polyclonal	IF (green)
Anti-Rabbit	A31572		Invitrogen	Donkey	Polyclonal	IF (red)
Anti-Mouse	A31570		Invitrogen	Donkey	Polyclonal	IF (red)

Validation

All of the primary antibodies could be used for WB assay. Among them, H3K4ac, H3K14ac, H3K18ac, H3K23ac, H3K36ac, H4k5ac, H4K8ac and H4K12ac antibodies could be used for ChIP assay and IF assay, they were used for WB assay in the manuscript (Supplementary figure 7a); H3K14ac antibody could be used for ChIP assay, IHC assay and IF assay and it was used for WB assay (figure 3a, d, f), ChIP assay (figure3c, e, g), IF assay (figure 3b and Supplementary figure 7d) and IHC assay (Supplementary figure 7c) in the manuscript; PCNA antibody was used for IF assay (Supplementary figure 2f); SP1 antibody was used for ChIP assay (figure5c, e and h); HDAC3 antibody was uesd for WB assay (figure 1b, c and supplementary figure1a), ChIP assay (figure 2g), IF

assay (figure 1a, d and supplementary figure1c and 2a, e, f), IHC assay (Supplementary figure 1b) and IP assay (figure4); HDAC1was used for WB assay (Supplementary figure1a) and ChIP assay (Supplementary figure3b); HDAC2 was used for WB assay (Supplementary figure1a); FOXO1 antibody was used for WB assay (figure4d); GAPDH antibody was used for all the WB assay as internal control reference.

Animals and other organisms

Policy information about studies involving animals; ARRIVE guidelines recommended for reporting animal research

Laboratory animals	The laboratory animal the study used was C57BL/6J female mice for the breeding and detection the phenotype of HDAC3cKO female mice. The three weeks old wild type C57BL/6J female mice were used for the cultures of ovarian follicles and COCs. The four weeks old wild type C57BL/6J female mice were used for in vitro maturation (IVM) and in vitro fertilization (IVF) of oocytes. The eight weeks old CD1 female mice were used for the surrogated mice.
Wild animals	The study did not involve wild animals.
Field-collected samples	The study did not involve samples collected from the field.
Ethics oversight	The experiments were performed in accordance with the principles and guidelines for the use of laboratory animals of China Agricultural University and approved by the Institutional Animal Care and Use Committee of China Agricultural University.

Note that full information on the approval of the study protocol must also be provided in the manuscript.
